# Spatial normalization of array-CGH data

**DOI:** 10.1186/1471-2105-7-264

**Published:** 2006-05-22

**Authors:** Pierre Neuvial, Philippe Hupé, Isabel Brito, Stéphane Liva, Élodie Manié, Caroline Brennetot, François Radvanyi, Alain Aurias, Emmanuel Barillot

**Affiliations:** 1 Institut Curie, Service de Bioinformatique, 26, rue d'Ulm, Paris, 75248 cedex 05, France; 2 Institut Curie, CNRS UMR 144, 26, rue d'Ulm, Paris, 75248 cedex 05, France; 3 Institut Curie, INSERM U509, 26, rue d'Ulm, Paris, 75248 cedex 05, France

## Abstract

**Background:**

Array-based comparative genomic hybridization (array-CGH) is a recently developed technique for analyzing changes in DNA copy number. As in all microarray analyses, normalization is required to correct for experimental artifacts while preserving the true biological signal. We investigated various sources of systematic variation in array-CGH data and identified two distinct types of spatial effect of no biological relevance as the predominant experimental artifacts: continuous spatial gradients and local spatial bias. Local spatial bias affects a large proportion of arrays, and has not previously been considered in array-CGH experiments.

**Results:**

We show that existing normalization techniques do not correct these spatial effects properly. We therefore developed an automatic method for the spatial normalization of array-CGH data. This method makes it possible to delineate and to eliminate and/or correct areas affected by spatial bias. It is based on the combination of a spatial segmentation algorithm called NEM (Neighborhood Expectation Maximization) and spatial trend estimation. We defined quality criteria for array-CGH data, demonstrating significant improvements in data quality with our method for three data sets coming from two different platforms (198, 175 and 26 BAC-arrays).

**Conclusion:**

We have designed an automatic algorithm for the spatial normalization of BAC CGH-array data, preventing the misinterpretation of experimental artifacts as biologically relevant outliers in the genomic profile. This algorithm is implemented in the R package MANOR (Micro-Array NORmalization), which is described at  and available from the Bioconductor site . It can also be tested on the CAPweb bioinformatics platform at .

## Background

Array-based comparative genomic hybridization (array-CGH) provides a quantitative measure of differences in copy number between two DNA samples [1]. The technique is typically applied to cancer studies because chromosome aberrations frequently occur during tumor progression [2]. Array-CGH facilitates the localization and identification of oncogenes and tumor suppressor genes, which are likely to be present in chromosomal regions gained and lost, respectively, in cancer cells.

Recent developments in the statistical analysis of array-CGH data have focused on high-level analysis, typically the identification of breakpoints from the genomic profile [3-7], rather than normalization. Most of the normalization techniques used to date for array-CGH data analysis have therefore involved the simple transposition of methods originally designed for expression data [8,9], correcting for differences in the labeling efficiency of the two dyes, spotting effects (block, row, column, or print-tip effects), and local or global intensity dependence of the ratios [10]. As far as we are aware, Khojasteh *et al*. [11] have reported the only method specific to CGH arrays.

Investigation of the systematic sources of variation in the array-CGH data studied showed that the effects affecting expression arrays were negligible with respect to spatial effects of two types. We describe here an algorithm for spatial normalization, which can also be combined with existing normalization methods for handling non-spatial artifacts. We will define and illustrate these two types of spatial effect, and show that such effects are not properly taken into account by traditional normalization techniques.

### Two distinct types of spatial artifact

The methods proposed here were originally developed for the analysis of bladder cancer data from tumors collected at Henri Mondor Hospital (Créteil, France) [12], analyzed by hybridization on CGH arrays (F. Radvanyi, D. Pinkel *et al*., unpublished results), including 2464 clones spotted at the University of California San Francisco (UCSF) [13]. They were then adapted to several data sets for CGH arrays produced and hybridized at the Institut Curie, including the breast cancer data (O. Delattre, A. Aurias *et al*., unpublished results) and the neuroblastoma data [14] (which is publicly available [15]) used to illustrate the technique.

**Figure 1:**
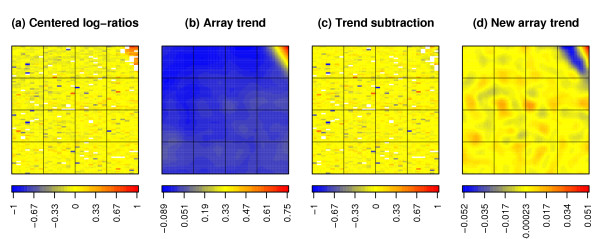
**The need for an image segmentation method**. An array with areas of local spatial bias (bladder cancer data): a straightforward trend correction method does not address the spatial effect appropriately. (a) Median-centered log-ratios; (b) spatial trend; (c) log-ratios after trend subtraction; (d) remaining spatial trend after subtraction (the color scale is not the same as in (b)). Colors are proportional to signal log-ratios; white dots correspond to missing values.

**Figure 2:**
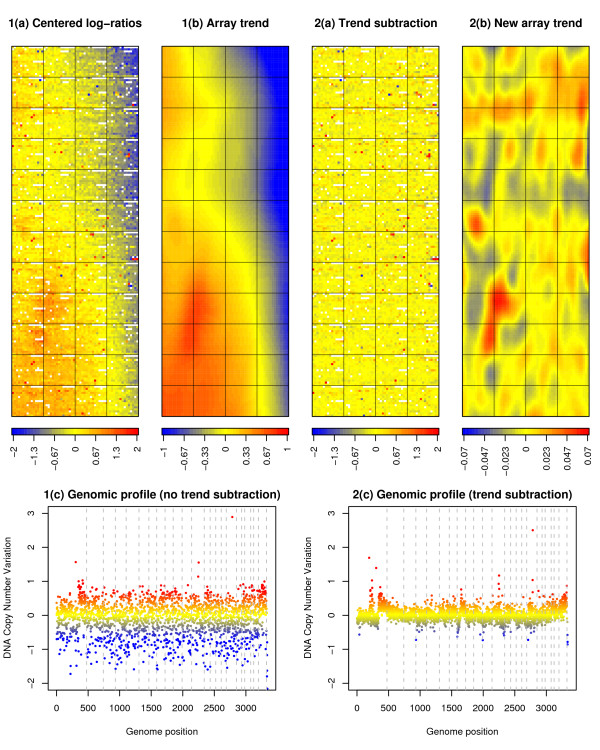
**Results of the gradient subtraction step (2dLoess) on a breast cancer array**. Correction of the spatial gradient of a breast cancer array: continuous spatial gradients are correctly taken into account by the proposed normalization method. 1(a) Median-centered log-ratios; 1(b) spatial trend; 1(c) genomic profile without spatial normalization; 2(a) corrected log-ratios; 2(b) spatial trend after correction (the color scale is not the same as in 1(b)); 2(c) genomic profile after spatial normalization. The vertical gray dashed lines indicate the separation between chromosomes.

We identified two types of spatial effect with fundamentally different natures: *local spatial bias *(Fig. 1(a)) and *continuous spatial gradients *(Fig. 2-1(a)):

#### Local spatial bias

**Figure 3:**
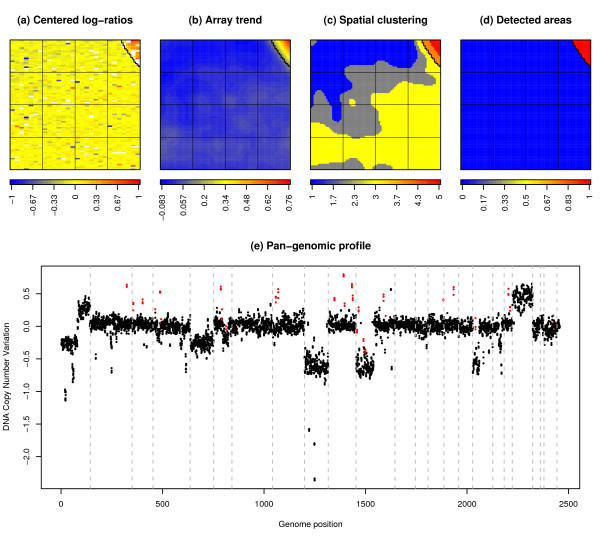
**Results of the proposed spatial segmentation method (seg) on a bladder cancer array**. Bladder cancer array with local spatial bias accurately detected by the proposed normalization method. (a) Median-centered log-ratios; (b) spatial trend; (c) spatial segmentation; (d) local spatial bias. The border of areas affected by local spatial bias that have been detected in panel (d) are reported on panels (a), (b) and (c) as a black step-function for easy interpretation; (e) genomic profile without spatial normalization (spots detected as local spatial artifacts are marked in red, and the vertical gray dashed lines indicate the separation between chromosomes).

The array image shows clusters of spots with a discrete signal shift, with the other spots of the array remaining unchanged. These clustered shifted spots on the array image (Fig. 1(a)) have no biological explanation, and correspond to outliers on genomic profiles (Fig. 3(e) and 6(e)). In the data sets studied here, this artifact was found to affect about half of all arrays. We describe it as *local *because it affects only limited areas of the array.

#### Continuous spatial gradient

The array image shows a smooth gradient in signal from one side of the slide to the other (Fig. 2-1(a)). This artifact leads to genomic profiles with high variability, even between regions with the same DNA copy number. When this effect is observed, it affects all spots to various degrees.

These two types of effect are experimental artifacts of non-biological origin:

- They occur on arrays designed such that neighboring spots on the array correspond to non-neighboring clones in the genome, so there is no obvious biological reason for the clustering of high (or low) signals on the array;

- They are frequently observed on control (normal tissue vs normal tissue) hybridizations, and even on background signals (see Figure 5 for illustration with the breast cancer data set).

The methods proposed are designed to remove or reduce these two types of spatial effect, while preserving the true biological signal.

### The need for a spatial segmentation method

The spatial effects described above cannot be attributed to spotting, for two reasons: firstly, they are not limited to array rows, columns or blocks; secondly, they are not reproducible from one array to another, even for arrays taken from batches of slides printed at the same time. Therefore, it is not possible to correct for them properly with the normalization methods generally used for expression arrays, in which "spatial" effects are captured only by row, column, or print-tip group effects. For a method to be appropriate, it must take into account the spatial structure of the array as a whole, and the arbitrary shape of these biased areas.

Several different studies have taken into account spatial effects in expression microarray data and have provided signal correction methods. For example, Workman *et al*. [16] defined a spatial gradient normalization method using a two-dimensional Gaussian function to estimate local background bias in a probe neighborhood. Baird *et al*. [17] proposed a mixed model for cDNA array data, using splines with spatial autocorrelation, assuming the existence of a one-step correlation between adjacent spots in a row or column. Colantuoni *et al*. [18] proposed a method for normalizing the element signal intensities to a mean intensity calculated locally across the surface of a DNA microarray. Others studies have combined intensity-dependent and spatially-dependent effects. Wilson *et al*. [19] have proposed fitting a single LOESS curve on the MA plot and then spatially smoothing the residuals using a median filter to estimate the spatial trend. Tarca *et al*. [20] proposed correcting intensity-dependent and spatially-dependent effects using a feed-forward neural network. Khojasteh *et al*. [11] have compared different CGH array data normalization methods and suggested that a three-step normalization that combines print-tip LOESS with spatial correction using moving median and microplate effect correction gave the best results.

These methods may be suitable for correcting continuous spatial gradients, but they were not designed to detect abrupt changes in signal value across the array, and therefore may not adequately handle local spatial bias: Figure 1 illustrates the need for a spatial *segmentation *method to handle such local spatial effects. From the median-centered log-ratios (a) we estimate a spatial trend (b) by two-dimensional LOESS regression [21,22]; subtracting this spatial trend from the raw values partially corrects the spatial effect (c), but the array trend after correction (d) demonstrates that the spatial effect is undercorrected at the inner border of the biased area, and overcorrected at the outer border, consistent with the observation that signal disturbances vary steeply at the border of the biased area. This systematic overcorrection or undercorrection may lead to misinterpretation in the corresponding genomic profile.

A similar type of spatial effect was reported for expression microarrays by Reimers *et al *[23]. For CGH arrays, this type of effect should be easier to detect and correct, as they have a much smaller range of signal ratio variation than expression microarrays. However, this smaller range necessitates a much greater measurement precision for array-CGH data.

We describe here a spatial segmentation algorithm for the automatic *delineation *and *elimination *of unreliable areas, facilitating the exclusion of local spatial bias from array-CGH data. This algorithm consists of three steps, which are explained in detail in the Methods section:

[step 1]: Estimation of a spatial trend on the array using two-dimensional LOESS regression [21,22]

[step 2]: Segmentation of the array into spatial areas with similar trend values using NEM, an unsupervised classification algorithm including spatial constraints [24,25]

[step 3]: Identification of the areas affected by spatial bias.

A wide variety of microarray techniques based on BACs, cDNAs or oligonucleotides (see [26] for a review) may be used to quantify changes in DNA copy number. From a technical aspect, our method could be applied to any of these microarray types, although we detected local spatial bias only on BAC arrays.

Therefore, we focused on this technology, which has also been the most widely used so far. We provide examples of the implementation of this method and illustrate its performance with three data sets collected on two CGH-array platforms:

- The first data set (bladder cancer data) was produced at the UCSF. In this data set, local spatial effects were observed on 57% of 198 arrays, with a median of 229 affected spots, and no visual evidence of spatial gradients;

- The two other data sets were produced at the Institut Curie, INSERM U509. They consist of a breast cancer data set, in which local spatial effects were observed on 45% of 175 arrays, with a median of 592 affected spots, and a neuroblastoma data set [14,15], with local spatial effects on 23% of 26 arrays, and a median of 551 affected spots.

### MANOR: an algorithm combining segmentation and signal correction

In addition to local spatial bias, we also frequently identified continuous spatial gradients, especially in breast cancer data set (Fig. 2-1(a)) and neuroblastoma data set. A straightforward way to correct for spatial gradients (Fig. 2-1(b)) is to subtract from the log-ratios an estimate of the spatial trend on the array (Fig. 2-2(a, b)). The first step of the spatial segmentation algorithm for detecting local spatial bias (step 1) provides such an estimate. This estimate is calculated using two-dimensional LOESS regression as explained in detail in the Methods section.

In many cases, the CGH arrays were affected by both types of spatial effect: local spatial effects and continuous spatial gradients. In practice, we do not know in advance what type of spatial effect affects a given array. Thus, we propose the following two-step approach:

1. run the spatial segmentation algorithm (*seg*) to identify potential areas of local spatial bias

2. correct spots not excluded during the first step for continuous spatial gradients (*2dLoess*).

This algorithm, implemented in the MANOR package, will be referred to as *seg+2dLoess *in the remainder of this article. The rationale underlying this two-step approach is that arrays affected by continuous spatial gradients only will not be detected as containing local spatial bias by the step *seg*, and will therefore be properly corrected by the step *2dLoess*. This two-step approach is suitable for the spatial normalization of data sets containing both types of spatial effect.

## Results and discussion

We have used our method for the spatial normalization of array-CGH data from two different platforms. In this section, we provide information about the practical implementation of the method on these two platforms, and quantitative results comparing our method to ten other normalization techniques. These compare the values of three quality criteria calculated after normalization of each array: the first, *sigma*, estimates the experimental variability between replicates, whereas the others, *smt *and *dyn*, evaluate quality in the context of the estimation of differences in DNA copy number between test and reference samples: *smt *quantifies the smoothness of the signal over the genome, and *dyn *assesses the dynamics of the signal, defined by the signal-to-noise ratio between gained and normal regions; these criteria are defined more formally and explained in detail in the Methods section.

To our knowledge, the ten normalization procedures used for the comparisons cover all the different types of approaches proposed so far and include the methods proposed by Tarca *et al*. [20], Yang *et al*. [10] and Khojasteh *et al*. [11]. These methods are detailed in the Methods section. For each normalization method, we calculated the three quality criteria for each array. When comparing two methods, we calculated a relative performance for each quality criterion, and assessed the significance of this performance using a Student's t-test, as explained in the Methods section. We show that our proposed method outperforms all previously published approaches for the three data sets.

### Application to data produced at UCSF

The bladder cancer data set to which our algorithm was applied concerns 198 arrays that were spotted and hybridized at UCSF. These arrays consist of 7392 spots, corresponding to 2464 clones – all of which are BACs (Bacterial Artificial Chromosomes) – with the following design:

- Neighboring clones in the genome are dispersed on the array – a necessary condition for distinguishing between spatial artifacts and real biological information;

- Each clone is replicated three times on the array, and the three replicated spots are adjacent, so a high level of consistency for the three corresponding ratios does not prove that there are no spatial effects.

For this data set, spatial normalization is the last step in the following comprehensive normalization process. After image analysis of the arrays with SPOT 2.0 software [27], we screened for low-quality spots: spots with a foreground reference signal (and foreground DAPI signal) less than 125% of the background reference signal (reference DAPI signal) were discarded, as were clones with a log-ratio standard deviation exceeding 0.1. Clones for which only one of the three replicates was retained after these steps were then also discarded.

Finally, we applied the proposed spatial normalization method *seg+2dLoess *as follows: the spatial segmentation *seg *was applied to the log-ratios of this filtered array, with *K *= 5 and *β *= 1 (see Methods for a definition of these parameters and a discussion of how to choose them), followed by the correction for continuous spatial gradients *2dLoess*.

#### Spatial normalization step

Our segmentation algorithm detected local spatial effects on 113 of 198 bladder cancer arrays (57%); the median proportion of biased areas on these arrays was 3.1%. Figure 3 (top) illustrates the successive steps of the algorithm, from centered log-ratios to array trend, spatial segmentation of the array, and finally the delineation of biased areas. Red dots on the corresponding genomic profile (Figure 3, bottom) correspond to the spots discarded during spatial normalization (on this figure, signal log-ratios have not yet been averaged by clone: *spot-level information *is displayed).

Figure 3 (bottom) illustrates the improvement in data quality achieved with our spatial normalization method: among the apparent outliers (i.e. clones with log-ratio values significantly different from the mean log-ratio value for the genomic region), it distinguished between experimental artifacts (red dots) and potentially biologically relevant outliers accounting for localized genomic amplifications.

#### Evaluation of the performance of the *seg+2dLoess *method

For each normalization method (11 methods including ours), we calculated the three quality criteria for each array and performed pairwise comparison of methods using the estimate and significance of their relative performance for each criterion, as explained in detail in the Methods section.

**Figure 4:**
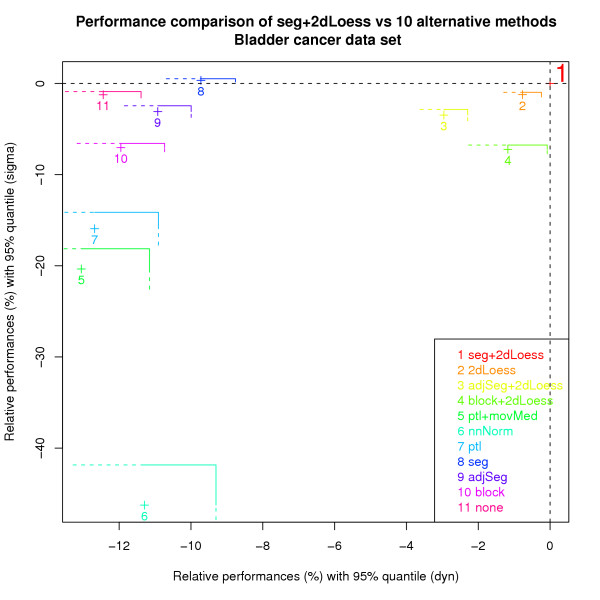
**The proposed method (seg+2dLoess) compares favorably to all other normalization methods – bladder cancer data set**. We compared the proposed method (*seg+2dLoess*) to ten methods for two quality criteria: *sigma *and *dyn*. Each color corresponds to the comparison of *seg+2dLoess *with a different method. The proposed method is taken as a reference (red point 1 at (0, 0)). For each method *i*, the cross indicates the mean relative performance (see methods section) of the data set for *dyn *(x axis) and in *sigma *(y axis), and the lines give the corresponding 95% quantile of relative performance. For *sigma *(*dyn*, respectively), the methods with a 95% quantile below (left to, respectively) the horizontal (vertical, respectively) dashed black line are significantly outperformed by our proposed method. Here *seg+2dLoess *significantly outperforms all methods for *dyn *and *sigma*, except *seg*, which performs slightly better for *sigma*. Methods 2, 3, and 4, which contain a gradient subtraction step using 2*dLoess*, perform the best against *seg+2dLoess*, as they cluster near the top-right corner of the image. However, *seg+2dLoess *still significantly outperformed these methods for both *sigma *and *dyn*.

Figure 4 shows the results of comparison of the ten methods with *seg+2dLoess*. For the *dyn *criterion, *seg+2dLoess *significantly outperformed all methods (with all *p*-values ≤ 0.039), and most significantly methods 5 to 11, that do not include the *2dLoess *step (with all *p*-values below 8.5 × 10^-18^). The *dyn *criterion is particularly important as it assesses the quality of copy number change detection. *seg+2dLoess *also gives significantly better results for the *sigma *criterion than all other methods (with all *p*-values below 1.1 × 10^-8^) except one: *seg *performs significantly better (*p *= 7.9 × 10^-4^) but the relative improvement has a limited amplitude (only 0.36%).

For the *smt *criterion, *seg+2dLoess *also significantly outperforms all methods (with all *p*-values below 8.1 × 10^-6^, except *block+2dLoess *for which *p *= 0.048).

Section 1 of the Additional file 1 shows similar plots to Figure 4, but for the *smt *and *dyn *criteria, and for the *smt *and *sigma *criteria. Tables 1 to 3 of the Additional files 2 and 3 summarize the results of all the pairwise comparisons of methods for the three quality criteria.

Taken together, these results show that the *seg+2dLoess *method outperforms its competitors for the bladder cancer data set.

### Application to data produced at Institut Curie, INSERM U 509

The Institut Curie, INSERM U509 has developed its own high-density CGH array; all steps in the production of these chips are performed in Institut Curie laboratories, including array spotting, DNA preparation, hybridization, scanning and image processing. The current version of the array contains 3342 clones, each of which is spotted at least three times on the array, giving a total of 10800 to 11520 spots (including controls).

This array was designed to facilitate distinction between relevant biological effects and experimental artifacts: "empty" spots and spots of water were included as controls, clone replicates were scattered over the array, and the positions of clones on the array are not correlated with their actual positions in the genome. A reliable ratio value can therefore be calculated even if one of the three replicates is flagged. The arrays were scanned using an Axon Genepix 4000b scanner, and images were processed with Genepix Pro 5.1.

We analyzed a breast cancer data set and a neuroblastoma data set from this platform.

For this platform, we applied the proposed spatial normalization method *seg+2dLoess *as follows: the spatial segmentation *seg *was applied to the Background signal as explained in the paragraph below, and the spatial gradients were corrected by *2dLoess *calculated over the log-ratios. A post-processing step that includes spot and clone screening was then applied (allowing us, for example, to discard spots having too low a signal-to-noise ratio, or with poor replicate consistency).

#### Detail of the spatial segmentation step

**Figure 5:**
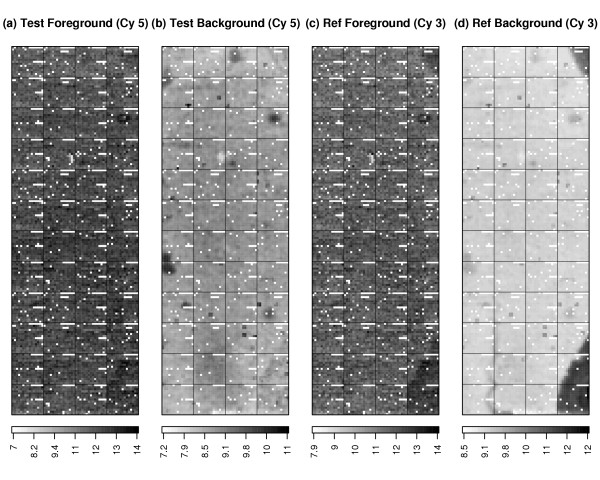
**Evidence of local spatial bias on foreground and background raw signals on a breast cancer array**. Log-ratios of the four raw signals of a breast cancer array: local spatial biases are easier to detect on a Cy3 background. (a) Test foreground; (b) test background; (c) reference foreground; (d) reference background. Gray-scale level is proportional to signal value.

Although we can correct the foreground signal for background intensity, a significant proportion of arrays still show localized spatial patterns that cannot be attributed to biological causes. Visual examination of spatial representations of the four signals (foreground and background intensities for test and reference signals) revealed that the bias was much clearer for the background signal of Cy3-labeled samples (Figure 5), which was not the case for bladder cancer data. We therefore applied the spatial segmentation method described above to the background signal of the Cy3 channel, with *K *= 7 and *β *= 1 (see Methods for a definition of these parameters and a discussion of how to choose them).

**Figure 6:**
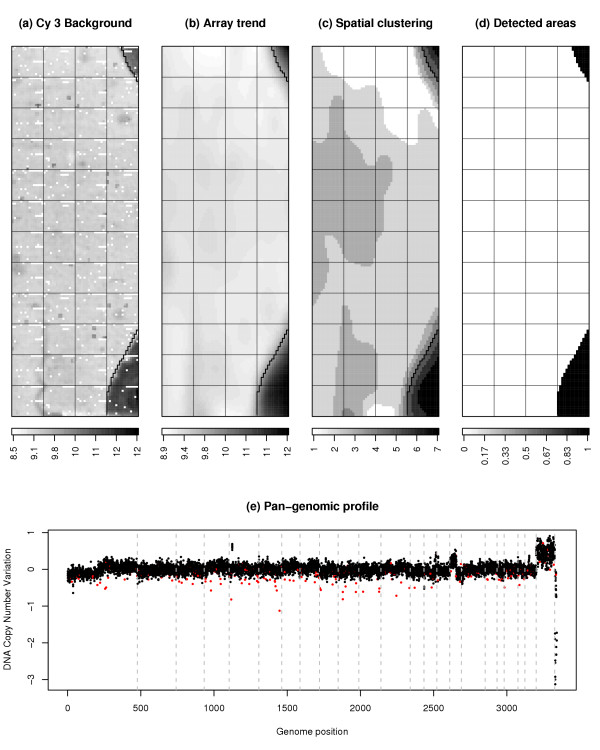
**Results of the local spatial normalization step (seg) on a breast cancer array**. Breast cancer array with local spatial bias accurately detected by the proposed normalization method. (a) Background signal log-ratios (Cy 3); (b) spatial trend; (c) spatial segmentation; (d) local spatial bias. The border of areas affected by local spatial bias that have been detected in panel (d) are reported on panels (a), (b) and (c) as a black step function for easy interpretation; (e) genomic profile without spatial normalization (spots detected as local spatial artifacts are marked in red, and the vertical gray dashed lines indicate the separation between chromosomes).

Biased areas of the CGH array are flagged and excluded from subsequent analysis. As clone replicates are not adjacent on the array, at least two of the three replicates generally remain after spatial bias correction, and a reliable ratio value can still be calculated. Figure 6 shows the results of this spatial segmentation step in the case of an array with local spatial bias but no spatial gradients.

#### Evaluation of the performance of the method *seg+2dLoess *

As for bladder cancer data, we calculated the three quality criteria for each normalization method and for each array for the breast cancer data set and the neuroblastoma data set. We then compared the methods paiwise using the estimate and significance of their relative performance for each criterion, as explained in detail in the Methods section.

**Figure 7:**
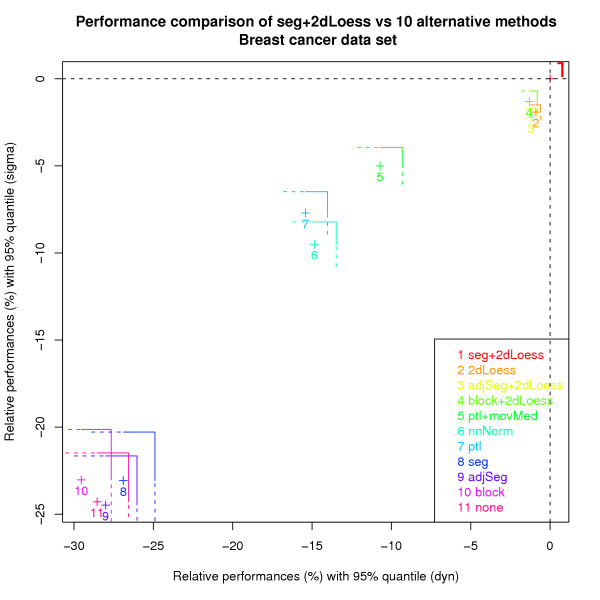
**The proposed method (seg+2dLoess) compares favorably to all other normalization methods – breast cancer data set**. We compared the proposed method (*seg+2dLoess*) to ten methods for two quality criteria: *sigma *and *dyn*. Each color corresponds to the comparison of *seg+2dLoess *with a different method. The proposed method is taken as a reference (red point 1 at (0, 0)). For each method *i*, the cross indicates the mean relative performance (see methods section) of the data set for *dyn *(x axis) and in *sigma *(y axis), and the lines give the corresponding 95% quantile of relative performance. For *sigma *(*dyn*, respectively), the methods with a 95% quantile below (left to, respectively) the horizontal (vertical, respectively) dashed black line are significantly outperformed by our proposed method. Here *seg+2dLoess *significantly outperforms all methods for *dyn *and *sigma*.

**Figure 8:**
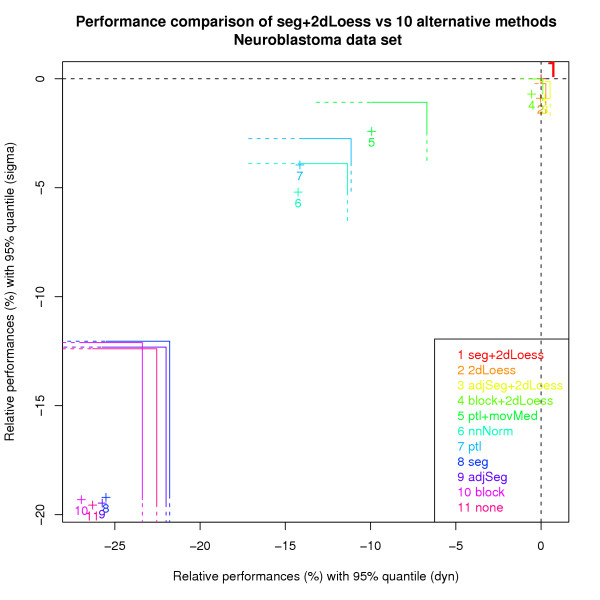
**The proposed method (seg+2dLoess) compares favorably to all other normalization methods – neuroblastoma data set**. We compared the proposed method (*seg+2dLoess*) to ten methods for two quality criteria: *sigma *and *dyn*. Each color corresponds to the comparison of *seg+2dLoess *with a different method. The proposed method is taken as a reference (red point 1 at (0,0)). For each method *i*, the cross indicates the mean relative performance (see methods section) of the data set for *dyn *(x axis) and in *sigma *(y axis), and the lines give the corresponding 95% quantile of relative performance. For *sigma *(*dyn*, respectively), the methods with a 95% quantile below (left to, respectively) the horizontal (vertical, respectively) dashed black line are significantly outperformed by our proposed method. Here *seg+2dLoess *significantly outperforms all methods for *dyn *and *sigma*, except those containing a gradient subtraction step with *2dLoess*.

Figures 7 and 8 show the results of comparing the ten methods with *seg+2dLoess *for the *dyn *and *sigma *criteria. *seg+2dLoess *significantly outperforms all other methods for the three criteria on the breast cancer data set (with all *p*-values below 2.3 × 10^-4^).

The neuroblastoma data set gives similar results: *seg+2dLoess *quality criteria are always better than those of the other methods, except for *dyn*, in which *adjSeg+2dLoess *is slightly better (0.22%) but not significantly so (*p *= 0.1). For *smt, seg+2dLoess *is only slightly better than *ptl+movMed *and the methods including the *2dLoess *step, but not significantly so for *adjSeg+2dLoess *and *ptl+movMed*. In these cases, the small size of the data set (26 arrays, 6 with local spatial bias) affects the statistical power.

Section 2 and 3 of the Additional file 1 and Tables 4 to 9 of the Additional files 2 and 3 detail and complement these results.

These results show that the *seg+2dLoess *method outperforms the other methods on the two data sets produced on the Institut Curie, INSERM U509 platform. The results also allow the methods to be ranked in terms of performance. Those methods that include a two-dimensional LOESS step are the highest ranked, with the methods proposed by [11,10] and [20], which all include some spatial processing, being next, and the other methods being the lowest ranked (see Figure 7 for example).

## Conclusion

We have designed an efficient and automated algorithm for the spatial normalization of BAC array-CGH data, and defined a set of parameters for CGH array data quality assessment. We have shown that our method significantly improves the quality of data from two different BAC-array platforms and outperforms other normalization techniques on three data sets.

The proposed algorithm is particularly suitable for correcting spatial effects not related to array design (row, column, or print-tip group effects): indeed, the arrays studied show two distinct types of such spatial effect (local spatial bias and continuous spatial gradients), which can simultaneously affect any given array. In such cases, using spatial trend correction after spatial segmentation helps to remove or reduce these two types of spatial effect, while preserving the true biological signal.

This method is original in the application of a segmentation algorithm for detecting and removing local spatial bias, preventing the misinterpretation of experimental artifacts as biologically relevant outliers in the genomic profile.

This method was developed for array-CGH experiments, and gave very good results. However, it can be applied to any microarray experiment having the same types of spatial effect.

## Availability and requirements

Our method is implemented in the R package MANOR (Micro-Array NORmalization) [28], which is available from the Bioconductor site [29]. It can also be tested on the CAPweb bioinformatics platform [30,31].

## Methods

In this section, we provide details of the segmentation method and the other normalization techniques used for comparison, and of the quality criteria proposed. We also discuss the choice of the two parameters of the segmentation algorithm: *K *and *β*.

### Description of the segmentation algorithm (seg)

The segmentation method consists of three steps:

[step 1]: Estimation of a spatial trend on the array using two-dimensional LOESS regression [21,22]

[step 2]: Segmentation of the array into spatial areas with similar trend values, using NEM, an unsupervised classification algorithm including spatial constraints [24,25]

[step 3]: Identification of the areas affected by spatial bias.

#### [step 1]: spatial trend estimation

We decided to carry out spatial segmentation based on an estimate of the spatial trend on the array, to optimize the robustness of segmentation. Furthermore, estimation of this trend makes it possible to replace missing values by interpolating the spatial trend.

The trend is estimated by means of a two-dimensional LOESS procedure with three iterative reweighting steps [21,22]. The local estimation is linear and the neighborhood taken into account to fit the local model corresponds to 3% of the total number of points. We use an iterative reweighting procedure to avoid outlier effects. Indeed, in the context of cancer studies, we are investigating changes in DNA copy number, and some clones displaying an amplification or a homozygous deletion may generate extreme but biologically meaningful values, which should not be interpreted as a local spatial bias.

When the spatial trend is estimated from the log-ratios, we first apply a basic correction to these log-ratios to prevent confusion between spatial artifacts and biologically relevant effects. For each chromosome arm, *centered *log-ratios are calculated as follows: the median of the corresponding log-ratio values is calculated and then subtracted from the initial values. The spatial trend is estimated from these centered log-ratios. This method helps to decrease the impact of true genomic aberrations on the detection of spatial trends in the data, particularly for samples with many, or large genomic alterations, as most of these alterations correspond to the gain or loss of whole chromosome arms.

#### [step 2]: spatial segmentation

This step aims to identify *K *clusters corresponding to spots with similar signal levels located close together geographically. This is achieved by Neighborhood Expectation Maximization (NEM) [24,25]. We assume that the data are drawn from a mixed Gaussian density function f(xi|Φ)=∑k=1Kpkfk(xi|θk)
 MathType@MTEF@5@5@+=feaafiart1ev1aaatCvAUfKttLearuWrP9MDH5MBPbIqV92AaeXatLxBI9gBaebbnrfifHhDYfgasaacH8akY=wiFfYdH8Gipec8Eeeu0xXdbba9frFj0=OqFfea0dXdd9vqai=hGuQ8kuc9pgc9s8qqaq=dirpe0xb9q8qiLsFr0=vr0=vr0dc8meaabaqaciaacaGaaeqabaqabeGadaaakeaacqWGMbGzcqGGOaakieqacqWF4baEdaWgaaWcbaGaemyAaKgabeaakiabcYha8HGabiab+z6agjabcMcaPiabg2da9maaqadabaGaemiCaa3aaSbaaSqaaiabdUgaRbqabaGccqWGMbGzdaWgaaWcbaGaem4AaSgabeaakiabcIcaOiab=Hha4naaBaaaleaacqWGPbqAaeqaaOGaeiiFaWhccmGae0hUde3aaSbaaSqaaiabdUgaRbqabaGccqGGPaqkaSqaaiabdUgaRjabg2da9iabigdaXaqaaiabdUealbqdcqGHris5aaaa@4CAE@ where *p*_*k *_are the proportions of the mixture model, *f*_*k *_(**x**_*i*_|***θ***_*k*_) denotes the density function of a Gaussian distribution with parameter ***θ***_*k *_= (***μ***_*k*_, **Σ**_*k*_) and **Φ **= {*p*_1_,..., *p*_*k *_, ***θ***_1_,..., ***θ***_*K*_} is the set of parameters to be estimated. The classical EM algorithm considers the following decomposition of the likelihood:

L(c,Φ)=∑i=1N∑k=1Kciklog⁡pkfk(xi|θk)−∑i=1N∑k=1Kciklog⁡cik     (1)
 MathType@MTEF@5@5@+=feaafiart1ev1aaatCvAUfKttLearuWrP9MDH5MBPbIqV92AaeXatLxBI9gBaebbnrfifHhDYfgasaacH8akY=wiFfYdH8Gipec8Eeeu0xXdbba9frFj0=OqFfea0dXdd9vqai=hGuQ8kuc9pgc9s8qqaq=dirpe0xb9q8qiLsFr0=vr0=vr0dc8meaabaqaciaacaGaaeqabaqabeGadaaakeaacqWGmbatcqGGOaakieqacqWFJbWycqGGSaaliiqacqGFMoGrcqGGPaqkcqGH9aqpdaaeWbqaamaaqahabaGaem4yam2aaSbaaSqaaiabdMgaPjabdUgaRbqabaaabaGaem4AaSMaeyypa0JaeGymaedabaGaem4saSeaniabggHiLdGccyGGSbaBcqGGVbWBcqGGNbWzcqWGWbaCdaWgaaWcbaGaem4AaSgabeaakiabdAgaMnaaBaaaleaacqWGRbWAaeqaaOGaeiikaGIae8hEaG3aaSbaaSqaaiabdMgaPbqabaGccqGG8baFiiWacqqF4oqCdaWgaaWcbaGaem4AaSgabeaakiabcMcaPiabgkHiTaWcbaGaemyAaKMaeyypa0JaeGymaedabaGaemOta4eaniabggHiLdGcdaaeWbqaamaaqahabaGaem4yam2aaSbaaSqaaiabdMgaPjabdUgaRbqabaaabaGaem4AaSMaeyypa0JaeGymaedabaGaem4saSeaniabggHiLdGccyGGSbaBcqGGVbWBcqGGNbWzcqWGJbWydaWgaaWcbaGaemyAaKMaem4AaSgabeaaaeaacqWGPbqAcqGH9aqpcqaIXaqmaeaacqWGobGta0GaeyyeIuoakiaaxMaacaWLjaWaaeWaaeaacqaIXaqmaiaawIcacaGLPaaaaaa@7811@

where

cik=pkfk(xi|θk)f(xi)  and  c=(cik)     (2)
 MathType@MTEF@5@5@+=feaafiart1ev1aaatCvAUfKttLearuWrP9MDH5MBPbIqV92AaeXatLxBI9gBaebbnrfifHhDYfgasaacH8akY=wiFfYdH8Gipec8Eeeu0xXdbba9frFj0=OqFfea0dXdd9vqai=hGuQ8kuc9pgc9s8qqaq=dirpe0xb9q8qiLsFr0=vr0=vr0dc8meaabaqaciaacaGaaeqabaqabeGadaaakeaacqWGJbWydaWgaaWcbaGaemyAaKMaem4AaSgabeaakiabg2da9maalaaabaGaemiCaa3aaSbaaSqaaiabdUgaRbqabaGccqWGMbGzdaWgaaWcbaGaem4AaSgabeaakiabcIcaOGqabiab=Hha4naaBaaaleaacqWGPbqAaeqaaOGaeiiFaWhcciGae4hUde3aaSbaaSqaaiabdUgaRbqabaGccqGGPaqkaeaacqWGMbGzcqGGOaakcqWF4baEdaWgaaWcbaGaemyAaKgabeaakiabcMcaPaaacaaMc8UaaGPaVlabbggaHjabb6gaUjabbsgaKjaaykW7caaMc8Uae83yamMaeyypa0JaeiikaGIaem4yam2aaSbaaSqaaiabdMgaPjabdUgaRbqabaGccqGGPaqkcaWLjaGaaCzcamaabmaabaGaeGOmaidacaGLOaGaayzkaaaaaa@5DB1@

In the mixture model context, [32] pointed out that the EM algorithm is formally equivalent to the alternative maximization of *L *(**c**, **Φ**) with respect to **c **("E" step) and with respect to **Φ **("M" step). The NEM algorithm is original in that it regularizes the likelihood by means of a term that takes into account the spatial dimension of the problem through the following adjacency matrix:

vij={1if i and j are neighbors0otherwise
 MathType@MTEF@5@5@+=feaafiart1ev1aaatCvAUfKttLearuWrP9MDH5MBPbIqV92AaeXatLxBI9gBaebbnrfifHhDYfgasaacH8akY=wiFfYdH8Gipec8Eeeu0xXdbba9frFj0=OqFfea0dXdd9vqai=hGuQ8kuc9pgc9s8qqaq=dirpe0xb9q8qiLsFr0=vr0=vr0dc8meaabaqaciaacaGaaeqabaqabeGadaaakeaacqWG2bGDdaWgaaWcbaGaemyAaKMaemOAaOgabeaakiabg2da9maaceaabaqbaeaabiGaaaqaaiabigdaXaqaaiabbMgaPjabbAgaMjabbccaGGqaciab=LgaPjabbccaGiabbggaHjabb6gaUjabbsgaKjabbccaGiab=PgaQjabbccaGiabbggaHjabbkhaYjabbwgaLjabbccaGiabb6gaUjabbwgaLjabbMgaPjabbEgaNjabbIgaOjabbkgaIjabb+gaVjabbkhaYjabbohaZbqaaiabicdaWaqaaiabb+gaVjabbsha0jabbIgaOjabbwgaLjabbkhaYjabbEha3jabbMgaPjabbohaZjabbwgaLbaaaiaawUhaaaaa@5F1A@

Here, the neighbors of a point located at coordinates (*l*, *m *) are the four points with the following coordinates: (*l *+1, *m*), (*l *- 1, *m*), (*l*, *m *- 1). We define the following quantity:

G(c)=12∑i=1N∑j=1N∑k=1Kcikcjkvij     (3)
 MathType@MTEF@5@5@+=feaafiart1ev1aaatCvAUfKttLearuWrP9MDH5MBPbIqV92AaeXatLxBI9gBaebbnrfifHhDYfgasaacH8akY=wiFfYdH8Gipec8Eeeu0xXdbba9frFj0=OqFfea0dXdd9vqai=hGuQ8kuc9pgc9s8qqaq=dirpe0xb9q8qiLsFr0=vr0=vr0dc8meaabaqaciaacaGaaeqabaqabeGadaaakeaacqWGhbWrcqGGOaakieqacqWFJbWycqGGPaqkcqGH9aqpdaWcaaqaaiabigdaXaqaaiabikdaYaaadaaeWbqaamaaqahabaWaaabCaeaacqWGJbWydaWgaaWcbaGaemyAaKMaem4AaSgabeaakiabdogaJnaaBaaaleaacqWGQbGAcqWGRbWAaeqaaOGaemODay3aaSbaaSqaaiabdMgaPjabdQgaQbqabaaabaGaem4AaSMaeyypa0JaeGymaedabaGaem4saSeaniabggHiLdaaleaacqWGQbGAcqGH9aqpcqaIXaqmaeaacqWGobGta0GaeyyeIuoaaSqaaiabdMgaPjabg2da9iabigdaXaqaaiabd6eaobqdcqGHris5aOGaaCzcaiaaxMaadaqadaqaaiabiodaZaGaayjkaiaawMcaaaaa@5883@

Thus, instead of maximizing *L *(**c**, **Φ **) in the E step, we maximize *L *(**c**, **Φ**) + *βG *(**c**). The value of *β *controls the weighting of the geographical context in the maximization. The M step remains unchanged.

#### [step 3]: elimination of local spatial bias

The basic idea is to remove from the array those spatial clusters with signal values significantly higher (or lower) than the unbiased areas of the array. We describe here the situation for positive spatial bias, but the idea can be adapted to negative bias. As local spatial biases cover a limited proportion of the array, we introduced a tuning parameter *p*_*max*_, which corresponds to the maximum proportion of the array image corresponding to local spatial bias. In our experiment, local spatial bias typically applies to less than one quarter of the array, so we used *p*_*max *_= 0.25.

After sorting the clusters identified by NEM by decreasing mean signal, we consider only those clusters with cumulative frequencies lower than *p*_*max *_to be potentially biased, making it possible to define a set of candidate clusters. The mean signal value of the remaining clusters is used as a reference value for the unbiased signal. Each candidate cluster with a mean signal differing from this reference value by more than a given threshold value is considered biased. The other candidates are considered unbiased, unless their mean signal is closer to that of the biased cluster than to that of the reference: such clusters are also considered biased. This threshold was chosen based on the cross-validation of arrays analyzed by experts.

### Comparison to other normalization methods

We compared the described methodology with other classical normalization methods. All these methods are listed below:

- *A print-tip group method:*

**block (block normalization): **we subtract off the row and column block median log-ratio values for each spot, and adds back the overall block median log-ratio value.

- *A print-tip group with intensity dependent effect method:*

**ptl (print-tip loess): **we apply the print-tip LOESS normalization [10] method using the marray R package (1.8.0 release, with default parameters) available from Bioconductor.

- *A spatial smoothing method:*

**2dLoess (correction of continuous spatial gradients): **a spatial trend is estimated by two-dimensional LOESS [21,22], which is then substrated from the log-ratio values.

- *Two spatial segmentation methods:*

**seg (segmentation of local spatial bias): **we apply the spatial segmentation algorithm described above to automatically eliminate the biased area.

**adjSeg (correction of local spatial bias): **we apply the spatial segmentation algorithm to automatically delineate the biased area. The median log-ratio value of such an area is then adjusted to the median log-ratio value of the unbiased area.

- *A method combining print-tip group and spatial smoothing:*

**block+2dLoess (block normalization and global correction): **we apply the *2dLoess *method on the normalized log-ratio values obtained with *block*.

- *Two methods combining intensity dependent effect and spatial smoothing:*

**nnNorm (neural network normalization): **we apply the normalization method described by Tarca *et al*. [20] using the nnNorm R package (1.5.1 release, with default parameters) available from Bioconductor. Briefly, this technique uses a neural network approach to correct the intensity-dependent and spatially-dependent effects.

**ptl+movMed (print-tip loess and moving median filter): **Khojasteh *et al*. [11] compared different normalization methods and suggested that combining the print-tip LOESS method with spatial correction (using a moving median calculated over a neighborhood of 11 rows by 11 columns) and microplate correction gave the best results. As the microplate information was not available in our data, we discarded the third step and only considered the print-tip LOESS and spatial correction.

- *Two methods combining spatial segmentation and spatial smoothing:*

**adjSeg+2dLoess (correction of local spatial bias and continuous spatial gradients): **we apply the *2dLoess *method on the normalized log-ratio values obtained with the *adjSeg *method.

**seg+2dLoess (local segmentation and correction of continuous spatial gradients): **we apply the *2dLoess *method on the log-ratio obtained with the *seg *method.

- Raw log-ratio values with no normalization (**none**).

### Array-CGH data quality assessment

#### Definition of quality criteria

Evaluation of the quality of the signal ratios of an array facilitates the comparison of different image analyses or normalization algorithms, and makes it possible to quantify the improvement achieved by each step of a given normalization algorithm. We define three criteria for assessing the quality of the analyzed array: the first addresses the issue of overall quality whereas the other two provide quality evaluations for the estimation of differences in DNA copy number between test and reference samples.

*sigma *The first item provides an estimate of experimental noise. We isolate each clone and calculate the standard deviation of the log-ratio of the corresponding replicates. *sigma *is defined as the median of these standard deviations: the smaller the value of *sigma*, the higher the quality of the array.

The other two criteria are calculated after detection of the altered (gained or lost) regions in the test sample. We used the GLAD algorithm, developed by Hupé *et al*. [4] for this purpose:

*smt *Within a given DNA copy number region, the ratios of contiguous clones should not differ considerably. The second quality criterion concerns the *smoothness *of the signal log-ratios within such a chromosomal region: signal smoothness is defined as the median absolute difference between log-ratios for contiguous normal clones. If *N *denotes the set of clones considered normal after DNA copy number estimation, we can calculate

*smt *= median_*n*∈*N*_|*x*_(*n*) _- *x*_(*n *-1)_|,

where *x*_(*n*) _is the value of the log-ratio at the *n*^th ^clone in genome order.

*dyn *The last criterion estimates the *dynamics *of DNA copy number variation between test and reference samples. We calculate the discrepancy between the median ratios of the regions considered "gained"(*G*) and "normal"(*N*) after DNA copy number estimation, and compare it with signal smoothness, as measured by *smt*:

dyn=mediang∈Gxg−mediann∈Nxnsmt
 MathType@MTEF@5@5@+=feaafiart1ev1aaatCvAUfKttLearuWrP9MDH5MBPbIqV92AaeXatLxBI9gBaebbnrfifHhDYfgasaacH8akY=wiFfYdH8Gipec8Eeeu0xXdbba9frFj0=OqFfea0dXdd9vqai=hGuQ8kuc9pgc9s8qqaq=dirpe0xb9q8qiLsFr0=vr0=vr0dc8meaabaqaciaacaGaaeqabaqabeGadaaakeaacqWGKbazcqWG5bqEcqWGUbGBcqGH9aqpdaWcaaqaaiabb2gaTjabbwgaLjabbsgaKjabbMgaPjabbggaHjabb6gaUnaaBaaaleaacqWGNbWzcqGHiiIZcqWGhbWraeqaaOGaemiEaG3aaSbaaSqaaiabdEgaNbqabaGccqGHsislcqqGTbqBcqqGLbqzcqqGKbazcqqGPbqAcqqGHbqycqqGUbGBdaWgaaWcbaGaemOBa4MaeyicI4SaemOta4eabeaakiabdIha4naaBaaaleaacqWGUbGBaeqaaaGcbaGaem4CamNaemyBa0MaemiDaqhaaaaa@55B5@

If no gained region is detected, we compare "normal" regions with "lost"(*L*) regions.

*smt *and *dyn *are not independent parameters and are anticorrelated. However, they quantify related but different ideas, as *smt *estimates the noise level after data normalization whereas *dyn *measures the ability to detect genome alterations after data normalization.

#### Paiwise comparison of quality criteria

These three criteria help us to decide which of two normalization methods gives the best results for a given array. In this pairwise comparison context, *smt *and *dyn *must be calculated with the same definition of *G*, *N*, and *L *regions for the two normalized arrays. We therefore define consensus *G*, *N*, and *L *regions associated with an array processed with two different normalization methods as the intersection of the two corresponding *G*, *N*, and *L *regions obtained using the two different normalization methods.

In order to test whether method *j *is better than method *i*, we defined a relative performance for each quality criterion as follows:



We calculated this relative performance for each array, and assessed its significance by testing the hypotheses ℋi,j
 MathType@MTEF@5@5@+=feaafiart1ev1aaatCvAUfKttLearuWrP9MDH5MBPbIqV92AaeXatLxBI9gBamrtHrhAL1wy0L2yHvtyaeHbnfgDOvwBHrxAJfwnaebbnrfifHhDYfgasaacH8akY=wiFfYdH8Gipec8Eeeu0xXdbba9frFj0=OqFfea0dXdd9vqai=hGuQ8kuc9pgc9s8qqaq=dirpe0xb9q8qiLsFr0=vr0=vr0dc8meaabaqaciaacaGaaeqabaWaaeGaeaaakeaaimaacqWFlecsdaWgaaWcbaacbiGae4xAaKgcbaGae0hlaWIae4NAaOgabeaaaaa@3B2C@: {*RP*^*qc*^(*i,j*) < 0} for each quality criterion *qc*, using a Student's unilateral t-test.

In figures 4, 7, and 8, we calculated relative performances *RP*(*seg+2dLoess*, *test*) where *test *corresponds to one of the ten other methods. Hence a *negative value *for *RP *(*seg+2dLoess*, *test*) indicates that our proposed method outperforms the *test *method.

### Parameter choice for the segmentation algorithm

The segmentation algorithm includes two parameters: the number *K *of clusters, and the regularization parameter *β*, which controls the weighting of geographic context in signal segmentation. Our experience suggests that the optimal choice of *K *and *β *may depend on the array-CGH technology used. We therefore provide guidelines for the choice of suitable parameters of the algorithm. We have investigated two different approaches to the choice of (*K*, *β*): incorporating a model selection criterion into the algorithm so that an optimal (*K*, *β*) can be chosen *for each array*, or developing a calibration method to help the user to find relevant sets of parameters for analyzing *a whole data set*. In this section, we discuss these two approaches and justify our choice of the second solution.

#### The difficulty finding optimal parameters on a per array basis

Choice of the number *K *of components in a mixture model can be addressed using model selection criteria. The basic idea is as follows: as the maximum likelihood estimator of the model increases mechanically with *K *(as model complexity increases with *K*), this method subtracts an increasing function of *K *from the likelihood of the model with *K *components, to prevent model overfitting. Many applications use the Akaike Information Criterion (AIC) or the Bayesian Information Criterion (BIC) for this purpose. However, in our framework, *K *and *β *must be chosen simultaneously, because *β *also affects the maximum likelihood estimator. As we have no information concerning the quantitative behavior of the maximum likelihood estimator with respect to *K *and *β *(this complex question is beyond the scope of this paper), the choice of an appropriate penalization remains arbitrary.

We also considered an approach involving the fitting of *K *using model selection criteria and cross-validating the choice of *β*, but this approach has major drawbacks: first, it strongly increases the complexity of the estimation process, making this method too time-consuming for use as a routine normalization method; second, it makes the normalization method difficult to interpret, because two arrays from the same platform will not be treated with the same parameters.

#### Guidelines for choosing relevant parameters for analyzing a new data set

Rather than searching for optimal (*K*, *β*) values for each array, we provide a calibration method making it possible to choose appropriate (*K*, *β*) values for each data set. The basic principle of the calibration method is comparison of the output of our algorithm run on different (*K*, *β*) pairs, taken from a pre-defined grid (e. g. *K *∈ {2,... 10} and *β *∈ {0.1,0.2,...2.0}).

We considered two different approaches to compare the results of the segmentations and to choose appropriate (*K*, *β*) values. The first approach involved choosing a (*K*, *β*) combination that optimizes quality criteria. The second involves expert assessment. An expert examines each array from a representative set and determines whether there is local spatial bias: he or she checks both the array image and the genomic profile to guarantee that the spatial effect is due to an experimental artifact rather than a biological effect. We then select the (*K*, *β*) combination that gives the best agreement between the expert decision and the algorithm decision. We call this second approach *expert assessment*. We found this second method simpler and more efficient than the first, for a number of reasons, outlined below.

In the first approach, quality criteria are calculated after normalization and DNA copy number assessment, so these three steps have to be carried out for each (*K*, *β*) combination. Therefore, although this method has the obvious advantage of not relying on expert assessment, it is time-consuming, and provides only indirect evaluations of the differences between pairs of parameters, which may make the results hard to interpret. Moreover, a much lower level of variation was observed in the values of quality criteria for different (*K*, *β*) combinations for a given array than between arrays, so we were unable to identify optimal (*K*, *β*) values with this method (data not shown).

In the second approach, we considered two different ways of performing the expert assessment: either identifying arrays displaying local spatial bias (qualitative assessment), or estimating the number of spots that should be discarded (quantitative assessment). We found quantitative assessment to be very poorly reproducible, with large differences between experts, and much more time-consuming than the qualitative method. Therefore, we adopted the qualitative method, which made possible the rapid expert assessment of a larger number of arrays, thus increasing the accuracy of parameter choice.

Based on the qualitative expert assessment of an entire data set or a subset of data, we compare, for each array, the decision of our algorithm (has the algorithm detected a local spatial bias?) with that of the expert. We then calculate the proportion of false positives and false negatives for each combination of the parameters *K *∈ {2,...10} and *β *∈ {0.1, 0.2,... 2.0}. Qualitative expert assessment remains highly variable (significant differences between experts), as a substantial proportion of arrays are difficult to classify. Nevertheless, all assessments show the same form of dependence in the error rate in (*K*, *β*), and lead to selection of the same parameters (data not shown).

**Figure 9:**
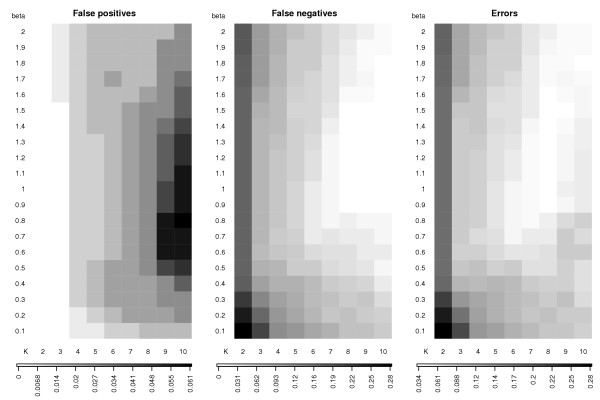
**Comparison between qualitative assessment and segmentation results with various (*K*, *β*) –breast cancer data set**. Thesegmentation algorithm is run with *K *∈ {2,...10} (*x *axis) and *β *∈ {0.1, 0.2,...2.0} (*y *axis) and compared with the expert assessment of the breast cancer data set. (a) False positive rate; (b) False negative rate; (c) Total error rate.

**Figure 10:**
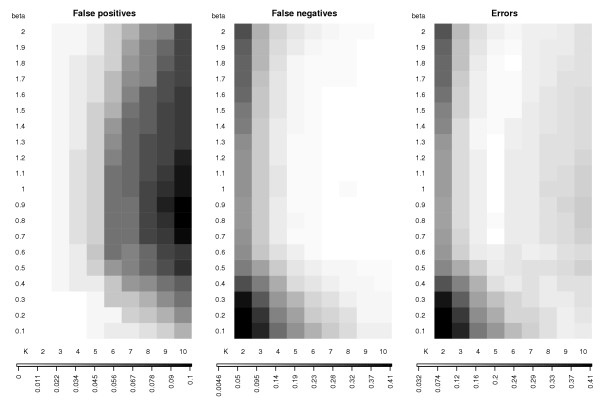
**Comparison between qualitative assessment and segmentation results with various (*K*, *β*) – bladder cancer data set**. The segmentation algorithm is run with *K *∈ {2,...10} (*x *axis) and *β *∈ {0.1, 0.2,...2.0} (*y *axis) and compared with the expert assessment of the breast cancer data set. (a) False positive rate; (b) False negative rate; (c) Total error rate.

For illustration, we use a subset of arrays on which two different expert assessments agree. The analysis is shown in Figure 9 for breast cancer data (134/179 arrays), and Figure 10 for bladder cancer data (169/198 arrays). False positives are arrays that experts identified as having no local spatial bias, but which were identified by the algorithm as having local spatial bias. False negatives are arrays that the expert considered to contain local spatial bias, and for which no such areas were reported by the algorithm. Roughly speaking, *K *controls cluster size, and *β *influences both the size and spatial coherence of the clusters. As *K *increases (with fixed *β*), clusters tend to shrink, leading to an increase in the mean signal value of the highest cluster, making it more likely that this cluster will be identified as a local spatial bias. For fixed *K*, the highest cluster is slightly more likely to be detected as local spatial bias for intermediate *β*, corresponding to an extreme cluster with high, homogenous values: for low *β *this cluster is often quite large and incorporates too small signal values, whereas for very high *β*, the geographic context is too strong, leading to a highest cluster with heterogeneous signal values.

Drawing figures such as Figure 9 or 10 for any new data set can facilitate the identification of relevant sets of parameters for the segmentation algorithm. In our case, they suggest values of *K *= 5 and *β *between 0.9 and 1.3 for bladder cancer data set, and *K *= 7 or 8 and *β *between 0.9 and 1.3 for breast cancer data set. We used *K *= 5, *β *= 1 for the bladder cancer data set, and *K *= 7, *β *= 1 for the breast cancer data set.

## Authors' contributions

PH and EB designed the study. PN and PH designed, coded and validated the spatial normalization algorithm. IB designed and coded the quality criteria. SL performed data integration. PH, PN, IB and EB drafted the manuscript. EM, CB, FR and AA performed the microarray experiments and validated the spatial normalization algorithm. FR, AA and EB supervised the study. All authors read and approved the final manuscript.

## Supplementary Material

Additional File 1 **Comparison of method seg+2dLoess with 10 alternative normalization methods**. We compared the method (*seg+2dLoess*) to ten methods for three quality criteria: *sigma*, *smt *and *dyn*. All images can be described as follows. Each color corresponds to the comparison of *seg+2dLoess *with a different method. The proposed method is taken as a reference (red point 1 at (0, 0)). For each method *i*, the cross indicates the mean relative performance on the data set for the two quality criteria compared, and the lines give the corresponding 95% quantile of the relative performance. The proposed method significantly outperforms, for the quality criterion shown in the y axis (at level 5%), all methods with a 95% quantile below the horizontal dashed black line. Similarly, the proposed method significantly outperformed, for the quality criterion shown in the x axis (at level 5%), all methods with a 95% quantile left of the vertical dashed black line. On most images, methods 2, 3, and 4, which contain a gradient subtraction step using 2*dLoess*, perform the best against *seg+2dLoess*, as they cluster near the top-right corner of the image. However, *seg+2dLoess *still significantly outperforms them for *sigma*, *smt *and *dyn*. Click here for file

Additional File 2 ***p*-values of the relative performances of 11 normalization methods**. We compare the results of 11 normalization methods on 3 data sets. Each table gives the significance levels of all pairwise comparisons between these 11 methods, for a given data set and a given quality measurement (*sigma*, *smt*, *dyn*). We calculated a relative performance for each array (as explained in the Methods section), and assessed its significance by testing the hypotheses ℋi,jqc
 MathType@MTEF@5@5@+=feaafiart1ev1aaatCvAUfKttLearuWrP9MDH5MBPbIqV92AaeXatLxBI9gBamrtHrhAL1wy0L2yHvtyaeHbnfgDOvwBHrxAJfwnaebbnrfifHhDYfgasaacH8akY=wiFfYdH8Gipec8Eeeu0xXdbba9frFj0=OqFfea0dXdd9vqai=hGuQ8kuc9pgc9s8qqaq=dirpe0xb9q8qiLsFr0=vr0=vr0dc8meaabaqaciaacaGaaeqabaWaaeGaeaaakeaaimaacqWFlecsdaqhaaWcbaacbiGae4xAaKMae4hlaWIae4NAaOgabaGae4xCaeNae43yamgaaaaa@3DD5@: {*RP^qc^*(*i*, *j*) < 0} for each quality criterion *qc*, using a Student's unilateral t-test. The *p*-value associated to ℋi,j
 MathType@MTEF@5@5@+=feaafiart1ev1aaatCvAUfKttLearuWrP9MDH5MBPbIqV92AaeXatLxBI9gBamrtHrhAL1wy0L2yHvtyaeHbnfgDOvwBHrxAJfwnaebbnrfifHhDYfgasaacH8akY=wiFfYdH8Gipec8Eeeu0xXdbba9frFj0=OqFfea0dXdd9vqai=hGuQ8kuc9pgc9s8qqaq=dirpe0xb9q8qiLsFr0=vr0=vr0dc8meaabaqaciaacaGaaeqabaWaaeGaeaaakeaaimaacqWFlecsdaWgaaWcbaacbiGae4xAaKgcbaGae0hlaWIae4NAaOgabeaaaaa@3B2C@ is reported in cell (*i*, *j*). Click here for file

Additional File 3 **Estimates of the relative performances of 11 normalization methods**. We compare the results of 11 normalization methods on 3 data sets. Each table gives the estimates of relative performance of all pairs of methods, for a given data set and a given quality measurement (*sigma*, *smt*, *dyn*). We calculated a relative performance for each array, and reported the mean value across all arrays of a given project in the following tables. Click here for file
