# Supplementary material for: Spatial normalization of array-CGH data
Source: BMC Bioinformatics. 2006 May 22;7:264. doi: 10.1186/1471-2105-7-264 (PMC1523216; doi:10.1186/1471-2105-7-264)
Supplement: Additional File 1 — Comparison of method seg+2dLoess with 10 alternative normalization methods. We compared the method (seg+2dLoess) to ten methods for three quality criteria: sigma, smt and dyn. All images can be described as follows. Each color corresponds to the comparison of seg+2dLoess with a different method. The proposed method is taken as a reference (red point 1 at (0, 0)). For each method i, the cross indicates the mean relative performance on the data set for the two quality criteria compared, and the lines give the corresponding 95% quantile of the relative performance. The proposed method significantly outperforms, for the quality criterion shown in the y axis (at level 5%), all methods with a 95% quantile below the horizontal dashed black line. Similarly, the proposed method significantly outperformed, for the quality criterion shown in the x axis (at level 5%), all methods with a 95% quantile left of the vertical dashed black line. On most images, methods 2, 3, and 4, which contain a gradient subtraction step using 2dLoess, perform the best against seg+2dLoess, as they cluster near the top-right corner of the image. However, seg+2dLoess still significantly outperforms them for sigma, smt and dyn. [file 1471-2105-7-264-S1.pdf]

Supplementary Materials 1: Comparison of method *seg+2dLoess* with 10 alternative normalization methods

Contents

1 Bladder cancer data set **2**
1.1 *smt* vs *sigma* 2
1.2 *smt* vs *dyn* 3

2 Breast cancer data set **4**
2.1 *smt* vs *sigma* 4
2.2 *smt* vs *dyn* 5

3 Neuroblastoma data set **6**
3.1 *smt* vs *sigma* 6
3.2 *smt* vs *dyn* 7

1 Bladder cancer data set

1.1 *smt* vs *sigma*

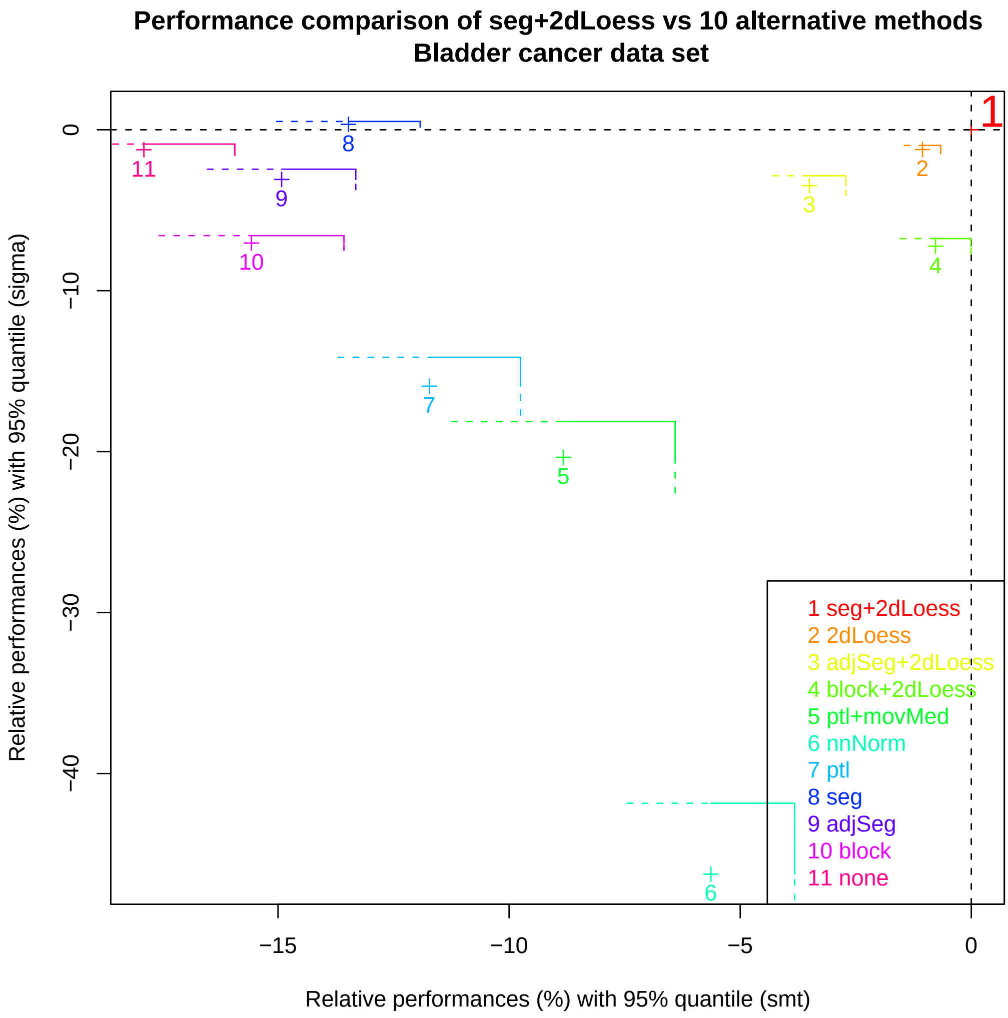

1.2 *smt* vs *dyn*

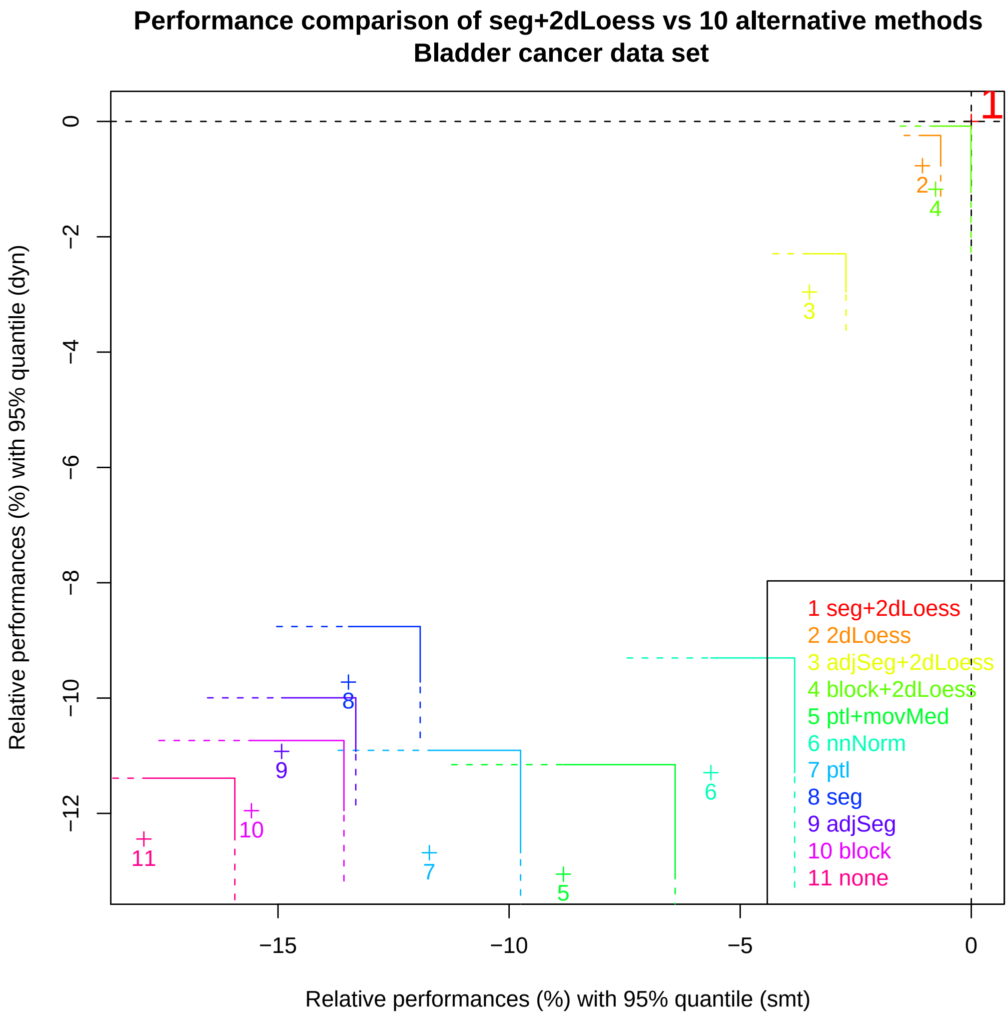

Performance comparison of seg+2dLoess vs 10 alternative methods
Bladder cancer data set
Relative performances (%) with 95% quantile (dyn)
Relative performances (%) with 95% quantile (smt)
0
−2
−4
−6
−8
−10
−12
−15
−10
−5
0
1 seg+2dLoess
2 2dLoess
3 adjSeg+2dLoess
4 block+2dLoess
5 ptl+movMed
6 nnNorm
7 ptl
8 seg
9 adjSeg
10 block
11 none

2 Breast cancer data set

2.1 *smt* vs *sigma*

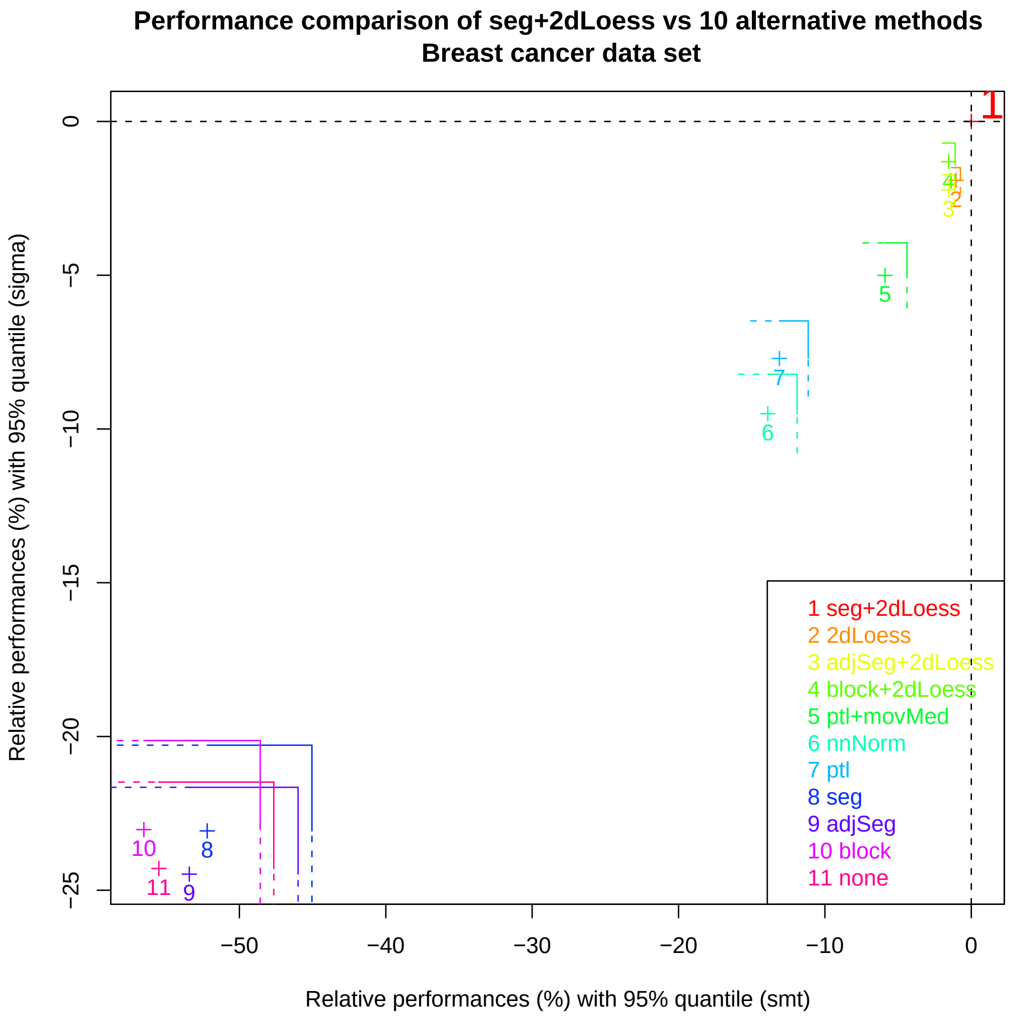

2.2 *smt* vs *dyn*

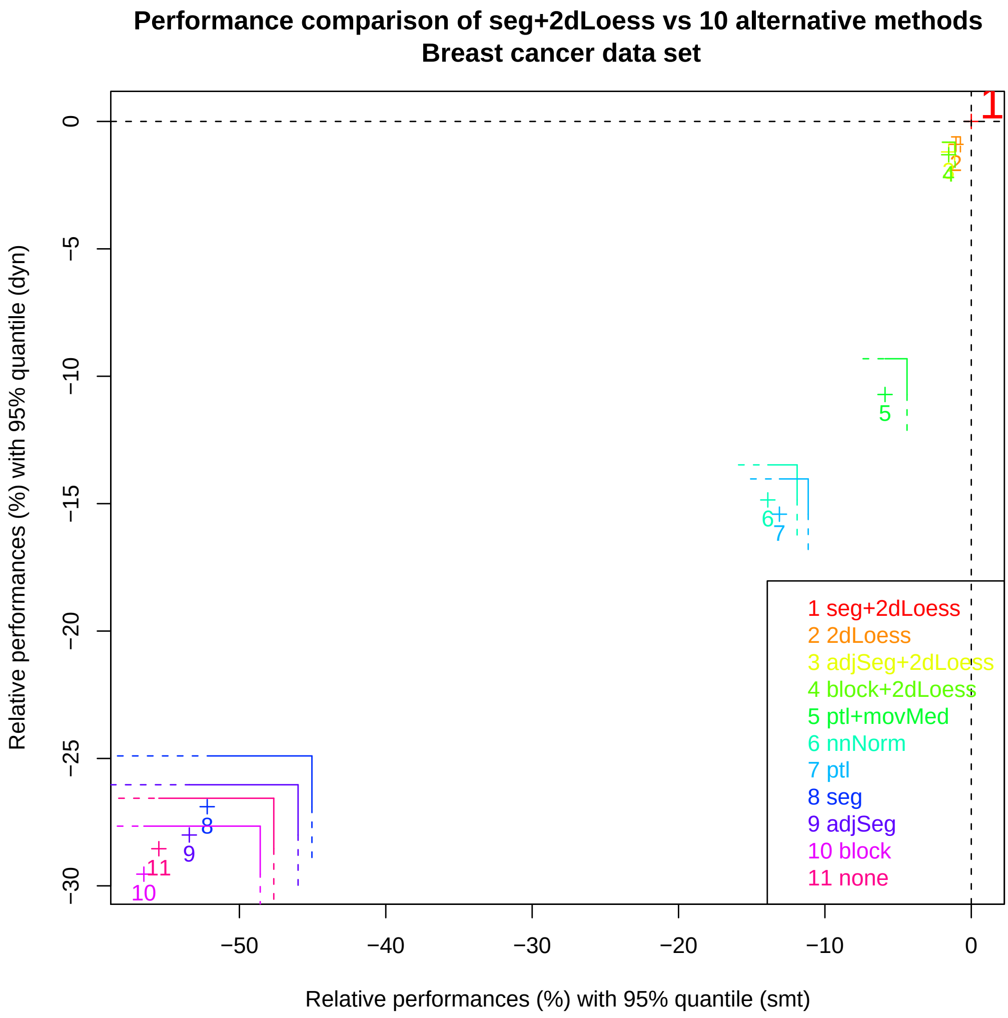
Performance comparison of seg+2dLoess vs 10 alternative methods
Breast cancer data set
Relative performances (%) with 95% quantile (dyn)
Relative performances (%) with 95% quantile (smt)
0
−5
−10
−15
−20
−25
−30
−50
−40
−30
−20
−10
0
1 seg+2dLoess
2 2dLoess
3 adjSeg+2dLoess
4 block+2dLoess
5 ptl+movMed
6 nnNorm
7 ptl
8 seg
9 adjSeg
10 block
11 none

3 Neuroblastoma data set

3.1 *smt* vs *sigma*

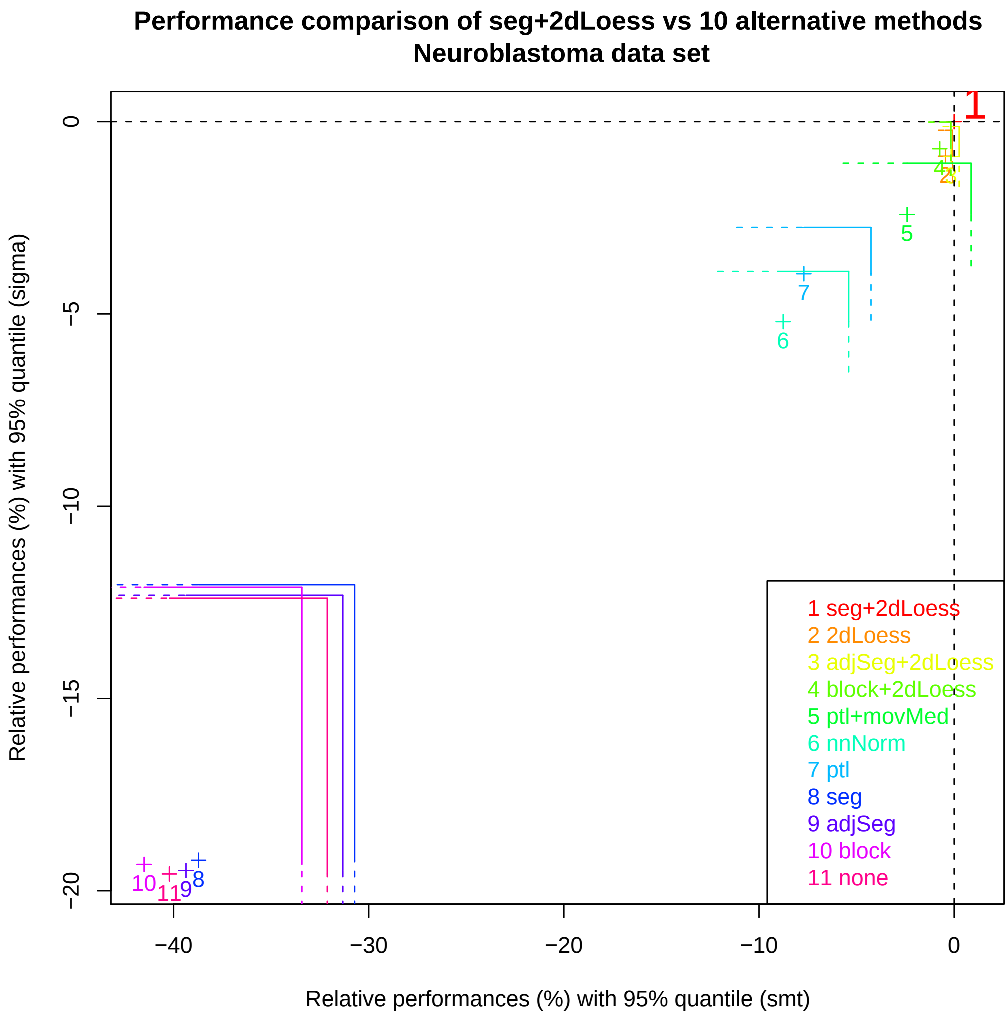

3.2 *smt* vs *dyn*

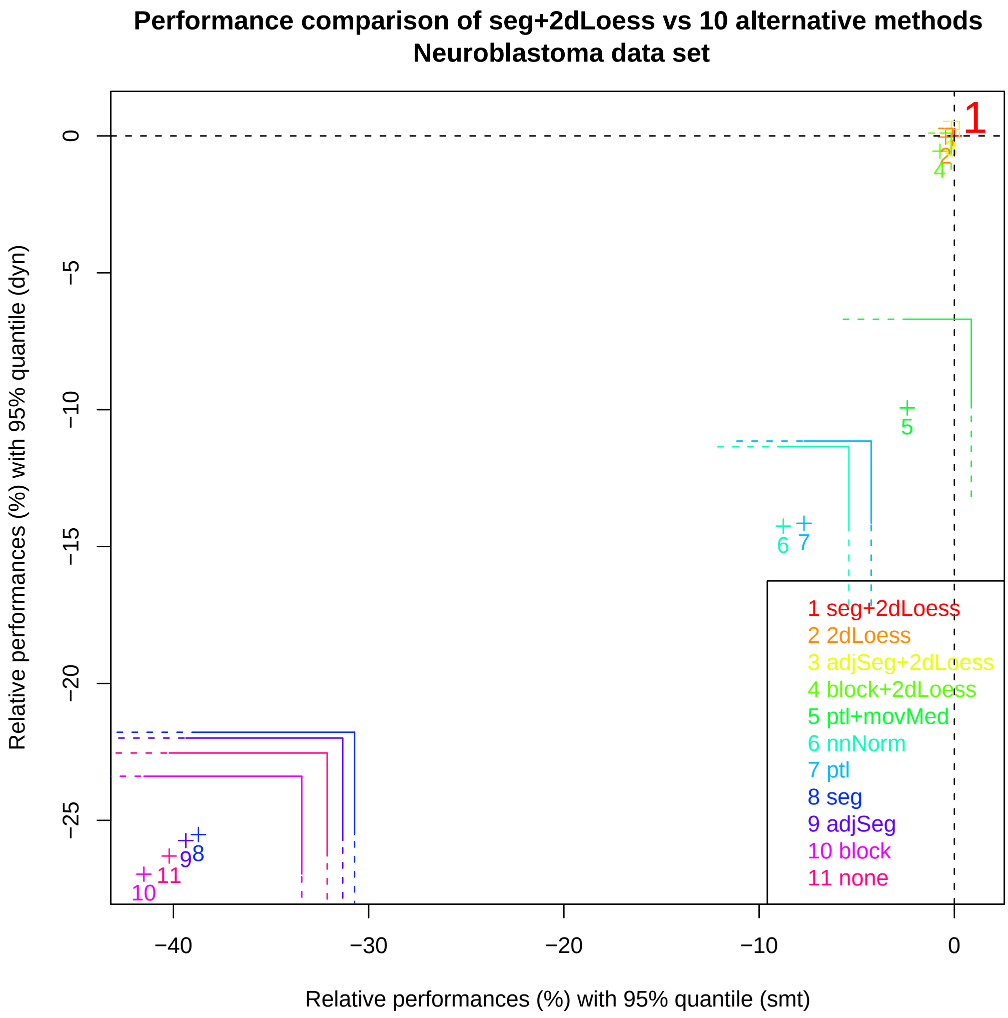

Performance comparison of seg+2dLoess vs 10 alternative methods
Neuroblastoma data set
Relative performances (%) with 95% quantile (dyn)
Relative performances (%) with 95% quantile (smt)
0
−5
−10
−15
−20
−25
−40
−30
−20
−10
0
1 seg+2dLoess
2 2dLoess
3 adjSeg+2dLoess
4 block+2dLoess
5 ptl+movMed
6 nnNorm
7 ptl
8 seg
9 adjSeg
10 block
11 none
