# Supplementary material for: Spatial normalization of array-CGH data
Source: BMC Bioinformatics. 2006 May 22;7:264. doi: 10.1186/1471-2105-7-264 (PMC1523216; doi:10.1186/1471-2105-7-264)
Supplement: Additional File 2 — p-values of the relative performances of 11 normalization methods. We compare the results of 11 normalization methods on 3 data sets. Each table gives the significance levels of all pairwise comparisons between these 11 methods, for a given data set and a given quality measurement (sigma, smt, dyn). We calculated a relative performance for each array (as explained in the Methods section), and assessed its significance by testing the hypotheses ℋi,jqc MathType@MTEF@5@5@+=feaafiart1ev1aaatCvAUfKttLearuWrP9MDH5MBPbIqV92AaeXatLxBI9gBamrtHrhAL1wy0L2yHvtyaeHbnfgDOvwBHrxAJfwnaebbnrfifHhDYfgasaacH8akY=wiFfYdH8Gipec8Eeeu0xXdbba9frFj0=OqFfea0dXdd9vqai=hGuQ8kuc9pgc9s8qqaq=dirpe0xb9q8qiLsFr0=vr0=vr0dc8meaabaqaciaacaGaaeqabaWaaeGaeaaakeaaimaacqWFlecsdaqhaaWcbaacbiGae4xAaKMae4hlaWIae4NAaOgabaGae4xCaeNae43yamgaaaaa@3DD5@: {RPqc(i, j) < 0} for each quality criterion qc, using a Student's unilateral t-test. The p-value associated to ℋi,j MathType@MTEF@5@5@+=feaafiart1ev1aaatCvAUfKttLearuWrP9MDH5MBPbIqV92AaeXatLxBI9gBamrtHrhAL1wy0L2yHvtyaeHbnfgDOvwBHrxAJfwnaebbnrfifHhDYfgasaacH8akY=wiFfYdH8Gipec8Eeeu0xXdbba9frFj0=OqFfea0dXdd9vqai=hGuQ8kuc9pgc9s8qqaq=dirpe0xb9q8qiLsFr0=vr0=vr0dc8meaabaqaciaacaGaaeqabaWaaeGaeaaakeaaimaacqWFlecsdaWgaaWcbaacbiGae4xAaKgcbaGae0hlaWIae4NAaOgabeaaaaa@3B2C@ is reported in cell (i, j). [file 1471-2105-7-264-S2.pdf]

Supplementary Materials 2:
# p-values of the relative performances of 11 normalization methods

Contents

1 Bladder cancer data set **2**

1.1 *sigma* 2

1.2 *smt* 3

1.3 *dyn* 4

2 Breast cancer data set **5**

2.1 *sigma* 5

2.2 *smt* 6

2.3 *dyn* 7

3 Neuroblastoma data set **8**

3.1 *sigma* 8

3.2 *smt* 9

3.3 *dyn* 10

1 Bladder cancer data set

1.1 *sigma*

	seg	seg+2dLoess	none	2dLoess	adjSeg	adjSeg+2dLoess	block	block+2dLoess	ptl	ptl+movMed	nnNorm
seg		1	1	1	1	1	1	1	1	1	1
seg+2dLoess	0.00079		1	1	1	1	1	1	1	1	1
none	1.6e−15	1.1e−08		0.48	1	1	1	1	1	1	1
2dLoess	4.2e−15	4.9e−14	0.44		1	1	1	1	1	1	1
adjSeg	7.3e−17	5.1e−14	4.5e−16	1.5e−11		1	1	1	1	1	1
adjSeg+2dLoess	1.3e−18	1.4e−17	1.2e−17	4.5e−17	5.4e−05		1	1	1	1	1
block	8.9e−57	4.2e−64	1.1e−58	5.3e−73	9.9e−31	1.2e−32		0.98	1	1	1
block+2dLoess	7.3e−52	1.8e−63	1.5e−47	1.8e−65	3e−25	2.8e−28	0.014		1	1	1
ptl	2.8e−31	1.3e−33	1.3e−28	4.6e−31	5.2e−25	3.7e−26	3.1e−16	1e−16		1	1
ptl+movMed	7.1e−33	3.3e−35	7.1e−31	3.5e−33	2.4e−28	1.2e−29	1.5e−21	2.5e−22	2.4e−35		1
nnNorm	3.7e−40	7.4e−42	2.3e−39	6.5e−41	2e−38	1.7e−39	8.9e−36	1.3e−36	2.2e−41	1.5e−39	

Table 1: sigma

1.2 *smt*

	seg+2dLoess	block+2dLoess	2dLoess	adjSeg+2dLoess	nnNorm	ptl+movMed	ptl	seg	block	adjSeg	none
seg+2dLoess		0.65	1	1	1	1	1	1	1	1	1
block+2dLoess	0.048		0.91	1	1	1	1	1	1	1	1
2dLoess	8.1e−06	0.057		1	1	1	1	1	1	1	1
adjSeg+2dLoess	3e−12	6.2e−06	5e−09		0.5	1	1	1	1	1	1
nnNorm	3.3e−07	2.4e−06	9.4e−05	0.044		1	1	1	1	1	1
ptl+movMed	3.9e−09	1e−11	3.2e−07	0.00029	2.8e−11		1	1	0.84	1	1
ptl	4.5e−19	3.6e−22	1.1e−17	2.2e−12	1.4e−19	1.4e−14		0.83	0.97	0.99	1
seg	1e−32	5.4e−30	2e−28	1.9e−20	4.9e−13	1.7e−09	0.00094		0.96	1	1
block	3.4e−28	5.3e−31	3.9e−27	9.1e−22	1.5e−13	1.3e−08	6e−05	0.0091		0.23	1
adjSeg	7.3e−36	5.3e−35	2.4e−33	2.2e−23	1.2e−15	3.4e−12	1e−05	4.2e−07	0.44		1
none	7.7e−35	3.9e−36	6.1e−34	6.2e−32	1.2e−19	1e−16	1.1e−11	5.4e−14	1.1e−12	1.7e−13	

Table 2: smt

1.3 *dyn*

	seg+2dLoess	2dLoess	block+2dLoess	adjSeg+2dLoess	seg	adjSeg	block	nnNorm	none	ptl+movMed	ptl
seg+2dLoess		1	1	1	1	1	1	1	1	1	1
2dLoess	0.0083		0.85	1	1	1	1	1	1	1	1
block+2dLoess	0.039	0.52		0.97	1	1	1	1	1	1	1
adjSeg+2dLoess	2.4e−12	0.001	0.31		1	1	1	1	1	1	1
seg	9.8e−40	2.1e−32	1.7e−13	1.9e−17		1	1	1	1	0.98	1
adjSeg	6.5e−48	9e−42	2.4e−18	6.7e−27	1.3e−06		0.98	1	1	1	1
block	2.2e−38	3.3e−35	4.8e−35	2.6e−27	0.00044	0.064		0.98	1	0.97	1
nnNorm	8.5e−18	2.3e−15	8.8e−16	6.9e−12	0.35	0.44	0.74		0.95	0.99	0.99
none	4.2e−48	4.8e−45	1.1e−22	1.3e−36	1.6e−15	6.6e−13	0.22	0.9		0.97	0.97
ptl+movMed	1.3e−23	1.4e−21	8e−22	1.1e−16	0.039	0.086	0.3	0.11	0.76		0.81
ptl	6.2e−25	2.6e−23	9.8e−25	2.9e−18	0.0081	0.056	0.092	0.17	0.64	0.57	

Table 3: dyn

2 Breast cancer data set

2.1 *sigma*

	seg+2dLoess	block+2dLoess	2dLoess	adjSeg+2dLoess	ptl+movMed	ptl	nnNorm	block	seg	none	adjSeg
seg+2dLoess		0.99	1	1	1	1	1	1	1	1	1
block+2dLoess	0.00023		0.99	1	1	1	1	1	1	1	1
2dLoess	6.2e−13	0.014		1	1	1	1	1	1	1	1
adjSeg+2dLoess	1.3e−12	0.0019	0.0022		1	1	1	1	1	1	1
ptl+movMed	2.2e−13	2.3e−13	5.5e−08	1e−06		1	1	1	1	1	1
ptl	2.1e−20	3.3e−22	7.8e−16	1.7e−14	6.2e−25		1	1	1	1	1
nnNorm	1.4e−25	1.2e−27	2.9e−21	7.4e−20	2.8e−27	4.4e−11		1	1	1	1
block	3.5e−28	5.8e−29	1.3e−26	6.4e−26	6.2e−27	1.1e−23	2.9e−21		0.48	0.99	1
seg	1e−29	7.4e−28	1.1e−27	5.6e−27	9.1e−25	4.2e−21	1.3e−18	0.24		1	1
none	1.7e−31	2.8e−30	4.1e−30	1.9e−29	1.2e−27	4.1e−24	1.1e−21	0.013	3.4e−08		0.96
adjSeg	1.4e−31	2.1e−30	3e−30	1.2e−29	1.1e−27	3.5e−24	9.8e−22	0.0084	1.1e−09	0.029	

Table 4: sigma

2.2 *smt*

	seg+2dLoess	2dLoess	adjSeg+2dLoess	block+2dLoess	ptl+movMed	ptl	nnNorm	seg	adjSeg	none	block
seg+2dLoess		1	1	1	1	1	1	1	1	1	1
2dLoess	2.4e−08		1	0.96	1	1	1	1	1	1	1
adjSeg+2dLoess	1.3e−09	0.0017		0.47	1	1	1	1	1	1	1
block+2dLoess	1.4e−08	0.017	0.38		1	1	1	1	1	1	1
ptl+movMed	4.7e−10	9.1e−08	1.3e−06	1.1e−06		1	1	1	1	1	1
ptl	7.5e−22	1.6e−20	7.6e−20	4.8e−20	2.5e−28		1	1	1	1	1
nnNorm	3.6e−23	4.3e−22	1.6e−21	2.3e−21	3.1e−31	0.00024		1	1	1	1
seg	7.5e−25	5e−23	2.3e−23	5.2e−23	4e−24	2e−21	1.7e−21		1	1	0.77
adjSeg	2.4e−24	7.7e−24	2.3e−24	1.1e−23	1.4e−25	4.6e−23	6.3e−24	1e−06		1	0.99
none	8.8e−24	7.3e−24	1.1e−24	2.3e−23	2.1e−25	2.1e−23	1.1e−23	3.5e−05	0.004		0.87
block	5.5e−24	1.7e−24	1.6e−24	4.5e−24	2.7e−26	1.5e−24	1.1e−24	0.0014	0.002	0.06	

Table 5: smt

2.3 *dyn*

	seg+2dLoess	2dLoess	adjSeg+2dLoess	block+2dLoess	ptl+movMed	nnNorm	ptl	seg	adjSeg	none	block
seg+2dLoess		1	1	1	1	1	1	1	1	1	1
2dLoess	5.3e−07		1	0.96	1	1	1	1	1	1	1
adjSeg+2dLoess	1.5e−07	0.0064		0.64	1	1	1	1	1	1	1
block+2dLoess	1.1e−05	0.1	0.53		1	1	1	1	1	1	1
ptl+movMed	2e−26	3.5e−23	1.4e−22	6.8e−25		1	1	1	1	1	1
nnNorm	7e−41	3.3e−39	4.3e−38	1.3e−40	1.7e−16		0.94	1	1	1	1
ptl	2.4e−42	9e−40	2.9e−38	2.7e−41	5.9e−22	0.13		1	1	1	1
seg	2.1e−52	2.2e−49	3.8e−50	1.2e−49	6.1e−23	3.6e−17	3.5e−16		1	1	1
adjSeg	2.8e−55	3.2e−53	1.3e−53	3.9e−52	3.8e−25	1.5e−19	6.4e−18	0.00078		0.99	1
none	4.6e−56	1.3e−54	1.6e−54	4.4e−54	9.9e−26	3e−20	4.1e−19	2.3e−05	0.0089		0.99
block	6.1e−61	1.1e−60	3.2e−59	1.2e−60	5e−33	5.2e−27	5.3e−25	0.17	0.0016	0.015	

Table 6: dyn

3 Neuroblastoma data set

3.1 *sigma*

	seg+2dLoess	block+2dLoess	adjSeg+2dLoess	2dLoess	ptl+movMed	ptl	nnNorm	seg	block	adjSeg	none
seg+2dLoess		0.95	0.97	0.98	1	1	1	1	1	1	1
block+2dLoess	0.048		0.95	0.98	0.99	1	1	1	1	1	1
adjSeg+2dLoess	0.029	0.049		0.48	0.99	1	1	1	1	1	1
2dLoess	0.016	0.019	0.51		0.98	1	1	1	1	1	1
ptl+movMed	0.0024	0.0052	0.012	0.014		1	1	1	1	1	1
ptl	4e−06	2.2e−06	5.1e−06	1.1e−05	7.3e−06		1	1	1	1	1
nnNorm	2e−07	1.7e−07	3.8e−07	7.7e−07	3.7e−07	2.9e−06		1	1	1	1
seg	5.5e−05	9.4e−05	0.00011	0.00011	0.00015	0.00031	0.00047		0.62	0.98	0.96
block	5.6e−05	9e−05	1e−04	1e−04	0.00014	0.00029	0.00044	0.37		0.84	0.95
adjSeg	4.7e−05	7.7e−05	8.6e−05	8.9e−05	0.00012	0.00025	0.00038	0.02	0.16		0.81
none	4.5e−05	7.4e−05	8.2e−05	8.5e−05	0.00012	0.00023	0.00036	0.044	0.051	0.19	

Table 7: sigma

3.2 *smt*

	seg+2dLoess	adjSeg+2dLoess	2dLoess	ptl+movMed	block+2dLoess	ptl	nnNorm	seg	adjSeg	none	block
seg+2dLoess		0.72	0.98	0.8	0.98	1	1	1	1	1	1
adjSeg+2dLoess	0.26		0.96	0.78	0.97	1	1	1	1	1	1
2dLoess	0.023	0.042		0.71	0.83	1	1	1	1	1	1
ptl+movMed	0.11	0.13	0.17		0.23	1	1	1	1	1	1
block+2dLoess	0.019	0.03	0.16	0.63		1	1	1	1	1	1
ptl	0.00039	0.00049	0.00081	0.00031	0.0011		0.91	1	1	1	1
nnNorm	7.6e−05	9.1e−05	0.00016	8.5e−05	0.00046	0.07		1	1	1	1
seg	6.4e−09	8.1e−09	9.6e−09	1.3e−07	1.6e−08	2.5e−08	1.4e−07		0.93	0.94	0.98
adjSeg	5.2e−09	6.1e−09	7.1e−09	1e−07	1.2e−08	1.6e−08	8.8e−08	0.076		0.92	0.96
none	3.9e−09	4.2e−09	5.1e−09	7.8e−08	8.4e−09	7.6e−09	4.4e−08	0.072	0.085		0.86
block	2.1e−09	2.4e−09	3.1e−09	2.3e−08	3.7e−09	4.5e−09	7.9e−09	0.02	0.034	0.12	

Table 8: smt

3.3 *dyn*

	adjSeg+2dLoess	seg+2dLoess	2dLoess	block+2dLoess	ptl+movMed	nnNorm	ptl	seg	adjSeg	none	block
adjSeg+2dLoess		0.9	0.97	0.99	1	1	1	1	1	1	1
seg+2dLoess	0.1		0.6	0.92	1	1	1	1	1	1	1
2dLoess	0.026	0.41		0.94	1	1	1	1	1	1	1
block+2dLoess	0.014	0.081	0.062		1	1	1	1	1	1	1
ptl+movMed	1e−05	1e−05	2e−05	1.3e−05		0.99	1	1	1	1	1
nnNorm	6e−09	4.8e−09	1.1e−08	7.8e−08	0.015		0.74	1	1	1	1
ptl	1.3e−08	1.1e−08	4.4e−08	3.5e−08	0.0014	0.32		1	1	1	1
seg	7.7e−12	6.7e−12	8.2e−12	8.2e−11	1.2e−05	3.1e−05	2.9e−05		0.93	0.9	0.96
adjSeg	6.5e−12	5.7e−12	7.1e−12	7.3e−11	9.8e−06	2.5e−05	2.2e−05	0.067		0.87	0.95
none	4.5e−12	4e−12	5.2e−12	5.5e−11	8.8e−06	1.4e−05	1.4e−05	0.093	0.12		0.87
block	8.9e−13	7.8e−13	1.2e−12	2.9e−12	7.1e−07	7.9e−07	2.1e−06	0.048	0.063	0.15	

Table 9: dyn
