# Supplementary material for: Spatial normalization of array-CGH data
Source: BMC Bioinformatics. 2006 May 22;7:264. doi: 10.1186/1471-2105-7-264 (PMC1523216; doi:10.1186/1471-2105-7-264)
Supplement: Additional File 3 — Estimates of the relative performances of 11 normalization methods. We compare the results of 11 normalization methods on 3 data sets. Each table gives the estimates of relative performance of all pairs of methods, for a given data set and a given quality measurement (sigma, smt, dyn). We calculated a relative performance for each array, and reported the mean value across all arrays of a given project in the following tables. [file 1471-2105-7-264-S3.pdf]

Supplementary Materials 3:
Estimates of the relative performances of 11 normalization methods

Contents

1 Bladder cancer data set **2**
1.1 *sigma* 2
1.2 *smt* 3
1.3 *dyn* 4

2 Breast cancer data set **5**
2.1 *sigma* 5
2.2 *smt* 6
2.3 *dyn* 7

3 Neuroblastoma data set **8**
3.1 *sigma* 8
3.2 *smt* 9
3.3 *dyn* 10

1 Bladder cancer data set

1.1 *sigma*

	seg	seg +2dLoess	none	2dLoess	adjSeg	adjSeg +2dLoess	block	block +2dLoess	ptl	ptl+movMed	nnNorm
seg		0.00334	0.015	0.015	0.031	0.0346	0.0676	0.0691	0.127	0.154	0.28
seg+2dLoess	−0.0036		0.0115	0.0117	0.0276	0.0314	0.0646	0.0662	0.125	0.152	0.279
none	−0.0158	−0.0124		−6.86e−05	0.0167	0.0204	0.0534	0.0549	0.114	0.141	0.269
2dLoess	−0.0159	−0.0123	−0.000175		0.0165	0.0203	0.0536	0.0552	0.114	0.142	0.271
adjSeg	−0.0344	−0.0309	−0.0178	−0.0179		0.00373	0.0368	0.0381	0.0985	0.127	0.257
adjSeg+2dLoess	−0.0385	−0.0348	−0.0218	−0.0217	−0.00393		0.0332	0.0348	0.0959	0.124	0.256
block	−0.0744	−0.0704	−0.0576	−0.0573	−0.0398	−0.0356		0.00172	0.0647	0.0942	0.231
block+2dLoess	−0.0765	−0.0724	−0.0598	−0.0593	−0.042	−0.0377	−0.00186		0.0635	0.0932	0.23
ptl	−0.165	−0.159	−0.146	−0.145	−0.126	−0.121	−0.0825	−0.08		0.0333	0.183
ptl+movMed	−0.21	−0.204	−0.19	−0.189	−0.169	−0.163	−0.123	−0.12	−0.0354		0.157
nnNorm	−0.471	−0.463	−0.447	−0.444	−0.421	−0.413	−0.363	−0.359	−0.249	−0.204	

Table 1: sigma

1.2 *smt*

	seg +2dLoess	block +2dLoess	2dLoess	adjSeg +2dLoess	nnNorm	ptl+movMed	ptl	seg	block	adjSeg	none
seg+2dLoess		0.0024	0.00926	0.0305	0.0361	0.0591	0.0879	0.109	0.12	0.12	0.139
block+2dLoess	−0.00773		0.0051	0.0249	0.032	0.0512	0.0826	0.1	0.111	0.112	0.131
2dLoess	−0.0106	−0.00932		0.0215	0.022	0.047	0.077	0.0977	0.109	0.109	0.128
adjSeg+2dLoess	−0.035	−0.0342	−0.0247		−4.37e−05	0.0248	0.0532	0.0792	0.0822	0.0851	0.104
nnNorm	−0.0563	−0.0495	−0.0397	−0.0179		0.0232	0.0543	0.0627	0.0685	0.0733	0.0959
ptl+movMed	−0.0882	−0.0682	−0.0729	−0.0487	−0.0268		0.0324	0.0431	0.0257	0.0546	0.0769
ptl	−0.117	−0.104	−0.101	−0.0708	−0.0647	−0.037		0.0104	0.0181	0.0226	0.0471
seg	−0.135	−0.124	−0.12	−0.1	−0.0954	−0.0707	−0.0308		0.00895	0.0101	0.0337
block	−0.156	−0.138	−0.139	−0.102	−0.103	−0.0728	−0.0357	−0.0152		−0.00405	0.0229
adjSeg	−0.149	−0.137	−0.133	−0.105	−0.106	−0.0813	−0.0402	−0.0111	−0.000632		0.0241
none	−0.179	−0.166	−0.163	−0.128	−0.137	−0.11	−0.0675	−0.0386	−0.0265	−0.0267	

Table 2: smt

1.3 *dyn*

	seg +2dLoess	2dLoess	block +2dLoess	adjSeg +2dLoess	seg	adjSeg	block	none	nnNorm	ptl	ptl+movMed
seg+2dLoess		0.00935	0.0185	0.0347	0.119	0.133	0.155	0.156	0.173	0.183	0.203
2dLoess	−0.00768		0.00468	0.0197	0.107	0.119	0.133	0.142	0.155	0.169	0.187
block+2dLoess	−0.0118	0.000366		0.0158	0.0928	0.105	0.12	0.127	0.15	0.16	0.162
adjSeg+2dLoess	−0.0296	−0.0147	−0.00377		0.0856	0.103	0.0997	0.106	0.136	0.141	0.171
seg	−0.0972	−0.0866	−0.0706	−0.0663		0.0117	0.0323	0.0363	0.0543	0.0635	0.049
adjSeg	−0.109	−0.0985	−0.0824	−0.0822	−0.0105		0.0193	0.0225	0.0458	0.0483	0.0557
block	−0.12	−0.105	−0.0971	−0.0806	−0.0206	−0.00898		0.014	0.0357	0.0432	0.0795
none	−0.124	−0.114	−0.099	−0.0886	−0.0317	−0.0204	−0.00671		0.0215	0.0259	0.0312
nnNorm	−0.113	−0.101	−0.1	−0.0866	−0.00595	−0.00215	0.00986	0.026		0.0186	0.0112
ptl	−0.127	−0.119	−0.116	−0.0984	−0.029	−0.0184	−0.0149	0.00446	−0.00724		0.000936
ptl+movMed	−0.131	−0.121	−0.113	−0.107	−0.0278	−0.0174	−0.00735	0.00956	−0.00626	0.00493	

Table 3: dyn

2 Breast cancer data set

2.1 *sigma*

	seg +2dLoess	block +2dLoess	2dLoess	adjSeg +2dLoess	ptl+movMed	ptl	nnNorm	block	seg	none	adjSeg
seg+2dLoess		0.0103	0.0178	0.0204	0.0412	0.0636	0.0787	0.161	0.165	0.173	0.174
block+2dLoess	−0.0131		0.00606	0.00879	0.0317	0.0546	0.0696	0.154	0.155	0.164	0.165
2dLoess	−0.0191	−0.00756		0.00275	0.0241	0.0471	0.0623	0.147	0.15	0.158	0.16
adjSeg+2dLoess	−0.0222	−0.0105	−0.00294		0.0212	0.0443	0.0595	0.144	0.147	0.156	0.157
ptl+movMed	−0.0501	−0.0361	−0.03	−0.0272		0.0239	0.0393	0.127	0.128	0.137	0.138
ptl	−0.0771	−0.0626	−0.0564	−0.0535	−0.0252		0.0158	0.107	0.107	0.116	0.117
nnNorm	−0.095	−0.0804	−0.0741	−0.0711	−0.0426	−0.0169		0.0924	0.0925	0.102	0.103
block	−0.23	−0.212	−0.206	−0.203	−0.169	−0.139	−0.12		−0.000228	0.00994	0.0112
seg	−0.231	−0.216	−0.207	−0.204	−0.173	−0.143	−0.124	−0.00486		0.00952	0.0109
none	−0.243	−0.228	−0.219	−0.215	−0.184	−0.154	−0.134	−0.0143	−0.0101		0.00132
adjSeg	−0.245	−0.229	−0.22	−0.217	−0.186	−0.155	−0.136	−0.0159	−0.0115	−0.00142	

Table 4: sigma

2.2 *smt*

	seg +2dLoess	2dLoess	adjSeg +2dLoess	block +2dLoess	ptl+movMed	ptl	nnNorm	seg	adjSeg	none	block
seg+2dLoess		0.00983	0.0143	0.0141	0.0447	0.102	0.108	0.291	0.296	0.303	0.309
2dLoess	−0.0105		0.00379	0.00336	0.0349	0.0935	0.101	0.279	0.289	0.296	0.303
adjSeg+2dLoess	−0.0154	−0.00411		−0.000155	0.0305	0.0904	0.0979	0.279	0.288	0.295	0.302
block+2dLoess	−0.0154	−0.00398	−0.000682		0.0295	0.0895	0.096	0.278	0.287	0.293	0.301
ptl+movMed	−0.0589	−0.0474	−0.0432	−0.0408		0.0617	0.0684	0.256	0.264	0.269	0.276
ptl	−0.131	−0.12	−0.116	−0.113	−0.0699		0.00781	0.207	0.217	0.223	0.231
nnNorm	−0.139	−0.128	−0.125	−0.122	−0.0779	−0.00893		0.199	0.211	0.216	0.224
seg	−0.522	−0.498	−0.495	−0.496	−0.446	−0.338	−0.324		0.008	0.016	0.00947
adjSeg	−0.534	−0.517	−0.51	−0.513	−0.459	−0.352	−0.338	−0.00855		0.00869	0.0126
none	−0.555	−0.534	−0.527	−0.531	−0.472	−0.363	−0.349	−0.0192	−0.0111		0.00407
block	−0.565	−0.545	−0.541	−0.54	−0.475	−0.37	−0.356	−0.0227	−0.0176	−0.00657	

Table 5: smt

2.3 *dyn*

	seg +2dLoess	2dLoess	adjSeg +2dLoess	block +2dLoess	ptl+movMed	nnNorm	ptl	seg	adjSeg	none	block
seg+2dLoess		0.00965	0.013	0.0147	0.138	0.196	0.204	0.451	0.482	0.496	0.514
2dLoess	−0.009		0.00399	0.00393	0.127	0.188	0.192	0.443	0.479	0.477	0.497
adjSeg+2dLoess	−0.012	−0.00361		0.000917	0.126	0.186	0.188	0.453	0.465	0.486	0.516
block+2dLoess	−0.0131	−0.00306	0.000227		0.123	0.183	0.187	0.441	0.475	0.476	0.492
ptl+movMed	−0.107	−0.0983	−0.097	−0.0969		0.0551	0.0627	0.329	0.34	0.35	0.36
nnNorm	−0.149	−0.143	−0.141	−0.141	−0.0475		0.00607	0.246	0.262	0.265	0.278
ptl	−0.154	−0.146	−0.143	−0.143	−0.0545	−0.00384		0.247	0.258	0.269	0.288
seg	−0.269	−0.263	−0.265	−0.262	−0.187	−0.146	−0.143		0.00935	0.0165	0.0264
adjSeg	−0.28	−0.275	−0.273	−0.273	−0.195	−0.157	−0.151	−0.00804		0.0113	0.0222
none	−0.285	−0.279	−0.28	−0.277	−0.2	−0.16	−0.156	−0.014	−0.00852		0.0115
block	−0.295	−0.291	−0.294	−0.289	−0.214	−0.175	−0.172	−0.0125	−0.016	−0.00848	

Table 6: dyn

3 Neuroblastoma data set

3.1 *sigma*

	seg +2dLoess	block +2dLoess	2dLoess	adjSeg +2dLoess	ptl+movMed	ptl	nnNorm	seg	block	adjSeg	none
seg+2dLoess		0.00661	0.00853	0.00852	0.0222	0.037	0.0482	0.142	0.142	0.144	0.144
block+2dLoess	−0.00705		0.00191	0.00195	0.0158	0.0306	0.0418	0.136	0.137	0.138	0.139
2dLoess	−0.00897	−0.00194		3.22e−05	0.0138	0.0287	0.0399	0.134	0.135	0.136	0.137
adjSeg+2dLoess	−0.00908	−0.00199	−5.67e−05		0.0138	0.0287	0.04	0.134	0.135	0.136	0.137
ptl+movMed	−0.0241	−0.0169	−0.015	−0.0149		0.0149	0.0263	0.123	0.124	0.125	0.126
ptl	−0.0396	−0.0323	−0.0303	−0.0302	−0.0153		0.0117	0.11	0.111	0.112	0.113
nnNorm	−0.052	−0.0447	−0.0427	−0.0426	−0.0275	−0.0119		0.1	0.101	0.102	0.103
seg	−0.192	−0.184	−0.182	−0.182	−0.163	−0.145	−0.131		0.000649	0.00226	0.00294
block	−0.193	−0.185	−0.183	−0.183	−0.164	−0.146	−0.131	−0.000769		0.00154	0.00224
adjSeg	−0.195	−0.187	−0.184	−0.184	−0.166	−0.147	−0.133	−0.00229	−0.0016		0.000693
none	−0.196	−0.188	−0.185	−0.185	−0.166	−0.148	−0.134	−0.00302	−0.00229	−0.000709	

Table 7: sigma

3.2 *smt*

	seg +2dLoess	adjSeg +2dLoess	2dLoess	block +2dLoess	ptl+movMed	ptl	nnNorm	seg	adjSeg	none	block
seg+2dLoess		0.0014	0.00434	0.00705	0.0153	0.0638	0.0735	0.262	0.265	0.269	0.276
adjSeg+2dLoess	−0.00154		0.00342	0.00638	0.0136	0.0619	0.0711	0.26	0.264	0.268	0.275
2dLoess	−0.00446	−0.00353		0.00258	0.0101	0.0584	0.0676	0.257	0.261	0.266	0.273
block+2dLoess	−0.00737	−0.0067	−0.00277		0.00597	0.0556	0.0626	0.253	0.257	0.262	0.27
ptl+movMed	−0.0241	−0.0223	−0.0186	−0.0138		0.0462	0.0529	0.243	0.247	0.251	0.263
ptl	−0.077	−0.0747	−0.0709	−0.0675	−0.0526		0.00879	0.212	0.216	0.221	0.229
nnNorm	−0.0876	−0.0846	−0.0806	−0.0754	−0.0601	−0.00991		0.201	0.204	0.21	0.222
seg	−0.387	−0.384	−0.379	−0.373	−0.366	−0.297	−0.28		0.0042	0.00967	0.017
adjSeg	−0.394	−0.391	−0.386	−0.38	−0.372	−0.303	−0.286	−0.00443		0.00551	0.013
none	−0.402	−0.4	−0.395	−0.389	−0.38	−0.311	−0.293	−0.0109	−0.00595		0.00721
block	−0.415	−0.413	−0.408	−0.402	−0.397	−0.323	−0.312	−0.019	−0.0145	−0.0084	

Table 8: smt

3.3 *dyn*

	adjSeg +2dLoess	seg +2dLoess	2dLoess	block +2dLoess	ptl+movMed	nnNorm	ptl	seg	adjSeg	none	block
adjSeg+2dLoess		0.00231	0.0038	0.0092	0.124	0.178	0.178	0.379	0.385	0.396	0.407
seg+2dLoess	−0.00223		0.00051	0.00598	0.123	0.178	0.177	0.378	0.382	0.393	0.404
2dLoess	−0.0037	−0.00042		0.0055	0.118	0.172	0.171	0.374	0.379	0.39	0.401
block+2dLoess	−0.00876	−0.00556	−0.00519		0.114	0.158	0.167	0.363	0.369	0.379	0.393
ptl+movMed	−0.1	−0.0994	−0.0953	−0.0934		0.0441	0.051	0.236	0.24	0.251	0.256
nnNorm	−0.142	−0.143	−0.138	−0.127	−0.0359		0.00524	0.18	0.184	0.194	0.206
ptl	−0.142	−0.141	−0.136	−0.134	−0.0437	−0.0037		0.18	0.184	0.193	0.205
seg	−0.255	−0.255	−0.253	−0.245	−0.162	−0.132	−0.132		0.00374	0.0104	0.0185
adjSeg	−0.258	−0.257	−0.256	−0.248	−0.164	−0.134	−0.134	−0.00358		0.00729	0.0159
none	−0.264	−0.263	−0.261	−0.254	−0.17	−0.141	−0.141	−0.00902	−0.00641		0.00938
block	−0.271	−0.27	−0.268	−0.263	−0.177	−0.153	−0.151	−0.0158	−0.0136	−0.0078	

Table 9: dyn
